# Psychometric properties of the health-related quality of life instrument with 8 items: a systematic review and meta-analysis

**DOI:** 10.1186/s12955-026-02494-z

**Published:** 2026-03-04

**Authors:** Tai-Kyung Lee, So-Young Lee, Eun Cho

**Affiliations:** College of Pharmacy, Sookmyung Women’s University, Cheongpa-ro 47-gil 100, Yongsan-gu, 04310 Seoul, Republic of Korea

**Keywords:** Health-related quality of life, HINT-8, Health status indicators, Psychometrics, Reproducibility of results

## Abstract

**Background:**

The Health-Related Quality of Life Instrument with 8 items (HINT-8) is a preference-based generic measure developed in 2014 for the Korean population. HINT-8 encompasses eight domains: climbing stairs, pain, vitality, working, depression, memory, sleep, and happiness. With the increasing use of HINT-8 in diverse research settings, a comprehensive synthesis is needed to clarify its psychometric performance and clinical utility. This study evaluated the properties of HINT-8 through a systematic review and meta-analysis.

**Methods:**

We systematically searched PubMed, KoreaMed, KMbase, KISS, and Google Scholar for studies published from January 2019 to December 2024 reporting HINT-8 outcomes. Data on HINT-8 index scores and domain-level outcomes were extracted for quantitative synthesis. Four pooled analyses were conducted: (1) construct validity using correlation with EQ-5D and SF-36, (2) reliability using Cohen’s kappa and intraclass correlation coefficients, (3) overall and domain-specific ceiling effects based on response distributions, and (4) subgroup specific HINT-8 index scores across demographic and clinical characteristics. Random-effects meta-analyses were performed, and statistical heterogeneity was assessed using I² and τ² statistics.

**Results:**

Among the 70 studies reporting HINT-8 outcomes, 23 contributed to the quantitative synthesis. Lower HINT-8 index scores were observed among female, older adults, and individuals with lower socioeconomic status compared with their respective reference groups. Correlation coefficients demonstrated expected construct validity with higher correlations for similar domains. Distinct HINT-8 domains such as vitality, working, memory and sleep showed low correlations with EQ-5D or SF-36, reflecting its unique construct coverage. Reliability assessed by Cohen’s kappa ranged from 0.237 to 0.521 across domains, while intraclass correlation coefficients indicated substantial agreement. HINT-8 showed lower ceiling effects (5.4% [95%CI: 2.3–8.6]) than EQ-5D (41.0% [25.4–56.6]), indicating better discriminatory ability. HINT-8 index values showed clear differences across various health conditions and demographic factors.

**Conclusion:**

With enriched domains, HINT-8 demonstrated sensitivity and utility as a generic measure for capturing diverse health statuses when applied with established tariff-derived index scores. As all included studies were conducted in Korea, further validation in diverse settings is warranted.

**Supplementary Information:**

The online version contains supplementary material available at 10.1186/s12955-026-02494-z.

## Introduction

Health-related quality of life (HRQoL) has emerged as a fundamental outcome measure in healthcare, reflecting a paradigm shift from disease-focused to patient-centered approaches [1]. By capturing patients’ subjective experiences and health status, HRQoL provides crucial evidence for evaluating the effectiveness of medical interventions and facilitate evidence-based decision-making in clinical practice [2–5].

Generic HRQoL instruments, such as the EuroQoL 5-Dimension (EQ-5D) and Short Form 36 (SF-36), have been widely adopted to compare health outcomes across diverse conditions and population groups [6, 7]. The preference-based measures enable the calculation of quality-adjusted life years and are essential tools for health economic evaluations and healthcare resource allocation decisions. Despite their widespread use and demonstrated psychometric properties, existing generic instruments have several methodological and cultural limitations that compromise their applicability in specific contexts. The EQ-5D, while extensively validated—including in the Korean population [8]—has pronounced ceiling effects and insufficient sensitivity for detecting health differences in relatively healthy populations [9–12]. Moreover, the development of HRQoL instruments predominantly in Western countries raises concerns about their cross-cultural validity and appropriateness for Asian populations [13]. These considerations are particularly relevant for preference-based measures, since health valuations and quality of life priorities vary across cultural contexts, underscoring the need for culturally appropriate HRQoL assessment tools.

In response, the Korea Disease Control and Prevention Agency developed the Health-Related Quality of Life Instrument with 8 Items (HINT-8) in 2014 as part of a national health policy initiative [14]. The development process involved a rigorous methodology, including preliminary validation studies with 892 Korean adults. Its construct validity and reliability were subsequently established through comparative analyses with the EQ-5D and SF-36 in a sample of 300 Korean adults [14].

The HINT-8 comprises eight health-related domains (*climbing stairs*,* pain*,* vitality*,* working*,* depression*,* memory*,* sleep*, and *happiness*) with four response levels each (no, mild, moderate, and severe), generating 65,536 possible health states. These domains are conceptually organized into four health dimensions: physical (*climbing stairs*,* pain*,* vitality*), social (*working*), mental (*depression*,* memory*,* sleep*), and positive (*happiness*). The instrument utilizes a one-week recall period and produces scores from 8 to 32, with higher scores indicating a poorer health status [14].

The preference-based value sets of HINT-8 were established through comprehensive valuation in 2017, employing standard gamble and visual analogue scale (VAS) methods with 1,000 Korean adults [15]. This valuation generated quality weights and a final tariff, resulting in index values ranging from 0.132 to 1.0, where higher values represent better health status [15]. In a development study targeting the general population, the eight domains of HINT-8 explained 47.6% of the variance in EQ-VAS scores compared with 33.5% for EQ-5D-3L, including reduced ceiling effects and enhanced discriminatory capacity [14].

Following its adoption in the Korea National Health and Nutrition Examination Survey (KNHANES) in 2019, the HINT-8 has been implemented biennially alongside the EQ-5D to assess population health status. The instrument has subsequently been utilized in diverse research applications, including studies of populations with chronic diseases, mental health assessments, and evaluations of medical interventions. However, despite the growing use of HINT-8 in research and policy contexts, comprehensive evaluations of its overall effectiveness and performance remain limited.

This study aims to provide a systematic and comprehensive evaluation of the HINT-8 through a meta-analysis of published research, focusing on its validity, reliability, and clinical utility as an HRQoL measure. By synthesizing the available evidence, this study seeks to elucidate its strengths, identify potential limitations or areas requiring refinement, and support its broader application in both research and clinical practice.

## Methods

This study was conducted in accordance with the Preferred Reporting Items for Systematic Reviews and Meta-Analyses (PRISMA) guidelines [16], and the protocol has been registered on the Open Science Framework (OSF) (https://doi.org/10.17605/OSF.IO/XGMWH). 

### Search strategies and study selection

Two reviewers independently conducted a comprehensive literature search to identify all published studies that utilized HINT-8 as an HRQoL measure across five databases: PubMed, KoreaMed, KMbase, Korean Studies Information Service System (KISS), and Google Scholar. The search was restricted to articles published between 2019 and 2024, corresponding to the period following the adoption of HINT-8 in the KNHANES. The search strategy incorporated both controlled vocabulary and free-text terms, including “HINT-8,” “HINT-eight,” and “Health-related quality of life instrument with 8 items”. Full database-specific search strings are provided in Supplementary Material 1. Language restrictions were applied to Korean and English publications only. Gray literature, conference abstracts, dissertations, and unpublished protocols were excluded. Any discrepancies in study selection or data extraction were resolved through discussion and consensus between the two reviewers. Although the search period began in 2019, the original development study of HINT-8 (2014) [14] and recent data from an insomnia study conducted by the authors^1^ were also included in the validation synthesis, to broaden the available evidence.

### Data extraction and management

For the studies that used the HINT-8 instrument, descriptive information—such as study characteristics (study name, design, and publication year), sample characteristics (sample size, demographic composition, and clinical conditions), and HINT-8-related outcomes (summation scores, individual domain scores, and index values where applicable)—was extracted into a structured Excel database by two reviewers independently. Studies were assigned to quantitative synthesis according to the outcomes they reported. For validation studies, psychometric indicators (correlation coefficients with other generic measures and reliability statistics) were extracted. For ceiling effect analyses, two types of data were extracted: (1) the proportion of participants reporting the best health profile (11111111 for HINT-8, 11111 for EQ-5D); and (2) the proportion of “1” (no problem) responses for each HINT-8 domain, where response distributions were available. To enable synthesis relevant for health economic evaluations and cross-population comparisons, studies reporting HINT-8 index values were further categorized. From these studies, index scores were extracted according to (1) sociodemographic factors and (2) disease categories.

### Quality and risk of bias assessment

The methodological quality of psychometric validation studies was assessed using the COnsensus-based Standards for the Selection of health Measurement INstruments (COSMIN) Risk of Bias checklist, and each measurement property was evaluated in accordance with the COSMIN methodology [17]. For observational studies, including cross-sectional or cohort designs, the Risk of Bias Assessment Tool for Nonrandomized Studies (RoBANS 2) were applied [18]. Quality assessment was conducted to characterize the overall methodological rigor of the included studies; however, sensitivity analyses based on study quality were not performed, as the primary objective of this study was to comprehensively evaluate the performance stability and measurement properties of the HINT-8 rather than to exclude studies based on methodological stringency. Publication bias was not assessed because each quantitative synthesis included fewer than ten studies, for which funnel plot asymmetry and associated statistical tests are considered unreliable.

### Data synthesis

Random-effects meta-analyses were performed using inverse-variance weighting. Between-study variance (τ²) was estimated using the restricted maximum likelihood (REML) method, and statistical heterogeneity was assessed using I² and Cochran’s Q test [19]. For studies reporting multiple index results (e.g., stratified by household type or age group), sample size-weighted averages were calculated to derive a single estimate per study prior to inclusion in the meta-analysis. To address potential overlap in datasets, particularly among studies using the KNHANES, all included studies were examined for overlapping survey years and identical target populations. When such overlap was identified, only one study per overlapping datasets was retained.

(1) Demographic and socioeconomic analysis.

To examine HINT-8 performance across demographic subgroups, index scores were pooled according to gender, age, education level, employment status, and household income. For each subgroup, mean index values, standard deviation, and sample size were collected and used for pooling. Due to variations in subgroup definitions across studies, standardized classifications were established. Age categories (over 65, 75, 80, 60 − 64, and 65 − 74) were consolidated into “aged over 60 years.” Educational statuses were grouped into “middle school”, “high school”, and “college or higher”. Household income was collapsed into “low”, “medium”, and “high”.

(2) Overall and domain-specific ceiling effects.

Pooled ceiling effects were estimated by aggregating the number of events and sample size across studies. Overall ceiling effects were calculated from the proportion of best health profiles, while domain-specific ceiling effects were synthesized using the proportion of “no problem” responses per item. Pooled proportions were compared with ceiling effects reported in the development study [14].

(3) Validity and reliability.

To assess the construct validity of the HINT-8, Spearman correlation coefficients were pooled for associations between each HINT-8 domain and the corresponding domains of the SF-36 and EQ-5D, as well as for correlations between the HINT-8 index and SF-36 scores. Pearson correlation coefficients were pooled for associations between the HINT-8 index and EQ-5D index. For the insomnia dataset from our ongoing study^1^, correlation coefficients were not originally reported but were calculated directly by the authors. All correlation coefficients were converted to Fisher’s z-values to stabilize variance, then converted back to correlation coefficients after pooling. Test-retest reliability was assessed by pooling Cohen’s kappa values. Intraclass correlation coefficients (ICC) results were synthesized narratively rather than meta-analyzed because the included studies did not report essential methodological details, including the specific ICC model and the exact number of days for the test-retest interval, which are necessary to ensure comparability across studies. Internal consistency indices such as Cronbach’s α were not used as reliability metrics because HINT-8 was conceptually developed as a multidimensional, formative instrument.

(4) Disease-specific score analysis.

HINT-8 index scores were pooled by disease categories including chronic respiratory and cardiovascular disease, cancer, arthritis, hypertension, type 2 diabetes (T2DM), dyslipidemia, gastroesophageal reflux disease (GERD), dizziness, and activity restriction, as well as mental health conditions such as stress, self-rated health status, depression, and generalized anxiety disorder (GAD). For the study reporting median and interquartile range, mean score and standard deviation were estimated using conversion methods [20].

All statistical analyses were conducted using the meta package in R version 4.4.1.

## Results

### Study characteristics

**Fig. 1:**
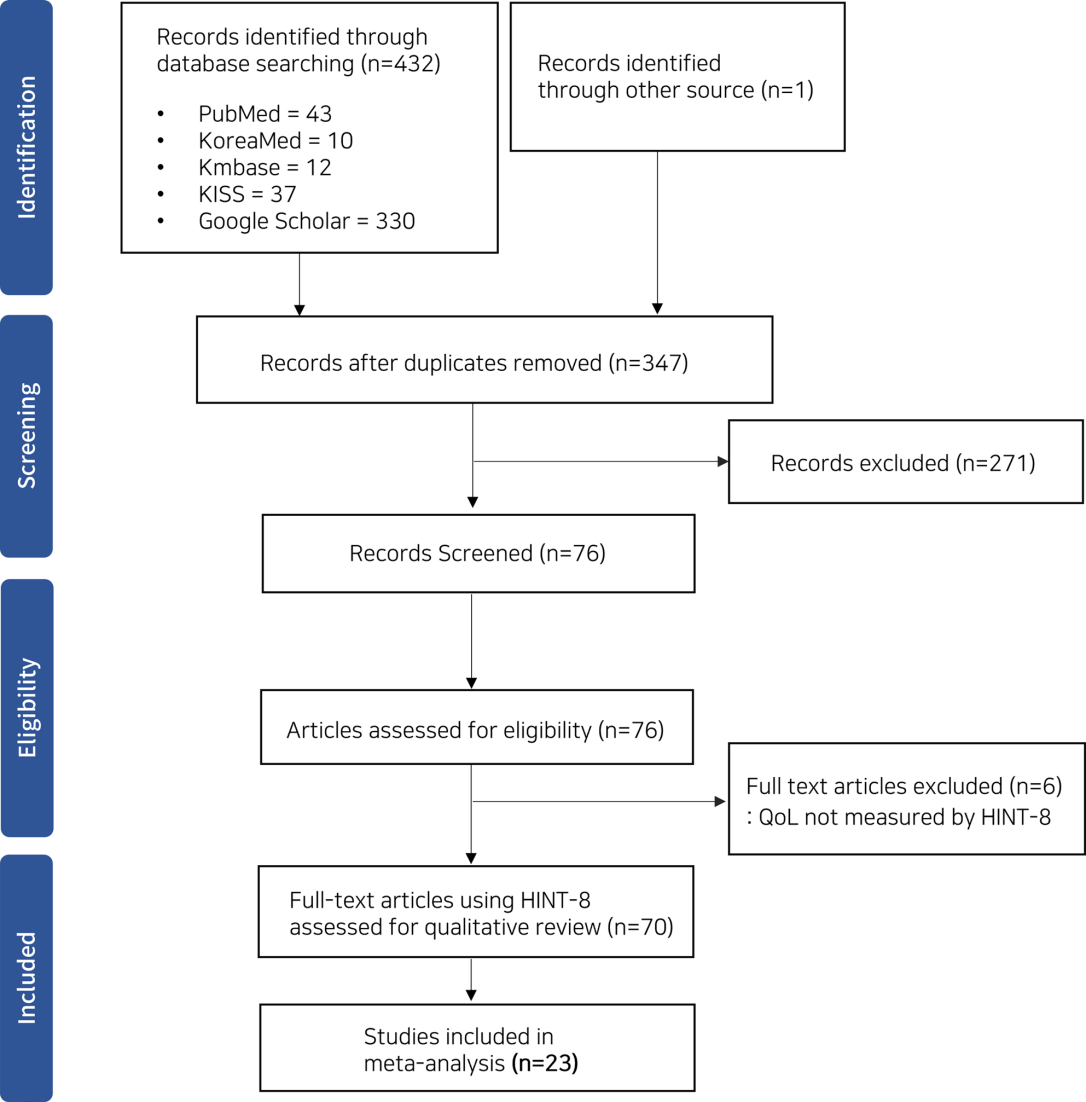
Flow diagram of study selection for HINT-8 studies

A total of 70 studies using HINT-8 were identified in the literature, which were briefly reviewed to understand the overall research landscape (Fig. 1). Most studies (*n* = 61, 87%) were clinical investigations, while five studies (7%) focused on HINT-8 validation using EQ-5D and SF-36. The remaining studies (*n* = 4, 6%) included mapping studies for HINT-8 to EQ-5D index conversion, internal structure analysis of HINT-8, qualitative investigations of HRQoL, and case report. The KNHANES served as the primary data source for the majority of studies (*n* = 48, 69%). In terms of HINT-8 utilization approaches, 29 studies (41%) employed HINT-8 index scores, 30 studies (43%) utilized total sum scores or domain-specific results, and eight studies (11%) focused on specific HINT-8 domains to evaluate particular dimensions of health status. Only 23 studies met the predefined eligibility criteria for quantitative synthesis (Supplementary Material 2). Studies included in each pooling category are summarized in Supplementary Material 3.

### Quality assessment

Based on the RoBANS 2 assessment, most included observational studies were rated as having a low risk of bias (Supplementary Material 4). The widespread use of KNHANES data, which employs standardized sampling and structured interviewing procedures, likely contributed to the generally low risk of measurement-related bias. However, several studies were rated as having a high risk of bias due to inadequate adjustment for potential confounders, particularly in multivariable analyses.

Based on the COSMIN framework, the risk of bias for PROM development and content validity was rated as “adequate”. Content validity was judged as “sufficient (+)”. Reliability showed a risk of bias ranging from “doubtful” to “inadequate”, but was still classified as “sufficient (+)”. However, the overall quality of evidence was graded as “low” due to differing measurement conditions and unreported ICC models. Hypothesis testing for construct validity demonstrated “very good” risk of bias and was assessed as “sufficient (+),” with the overall quality of evidence graded as “high” (Supplementary Material 5).

### HINT-8 values for general populations by demographic factors

**Fig. 2:**
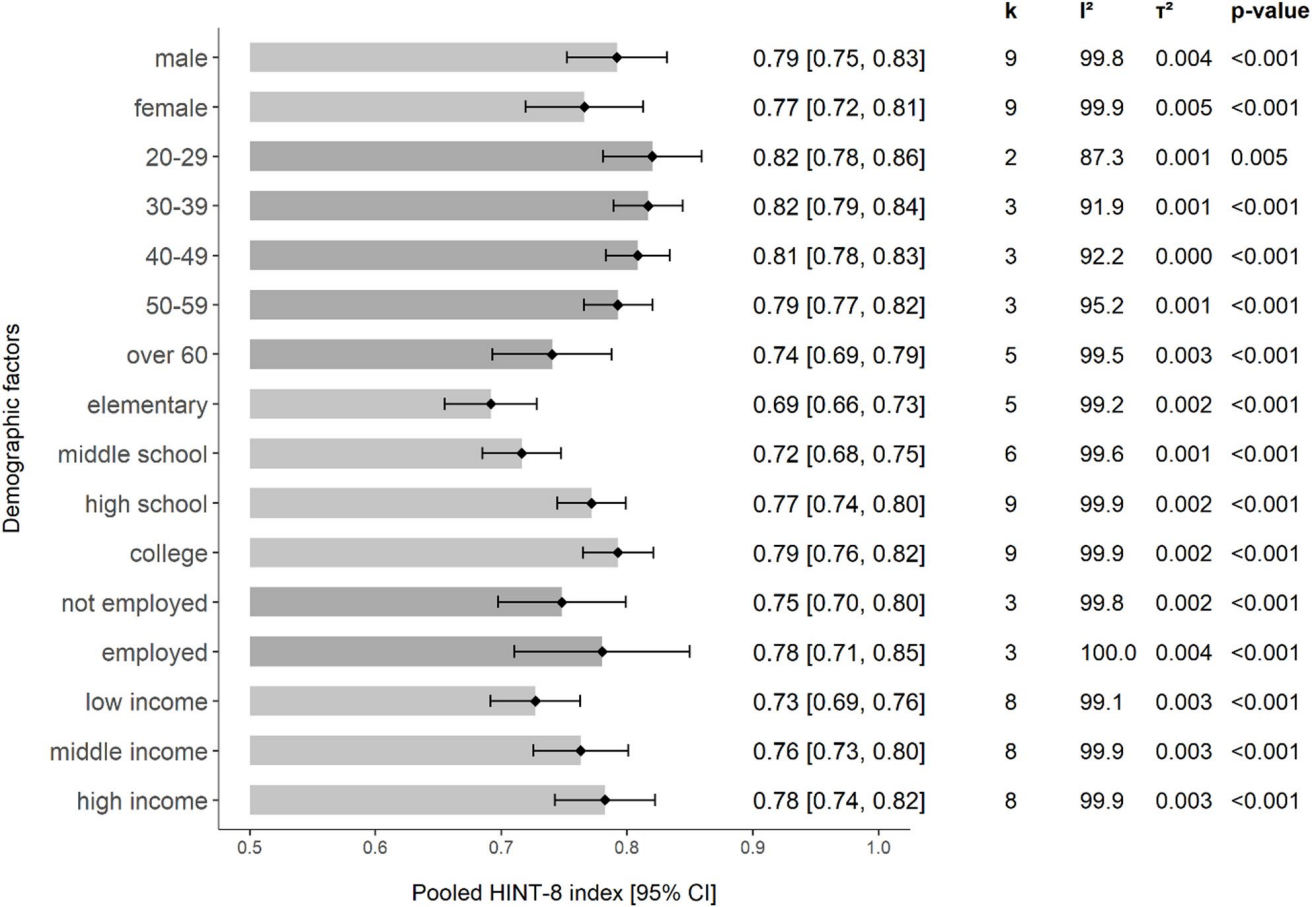
Pooled HINT-8 index values of general populations by demographic factors from 11 studies. Note. Random-effects models were used for all pooled estimates; studies with a standard deviation of 0 were excluded from pooling; k = number of studies included

Pooled mean HINT-8 index scores across various demographic subgroups from 11 studies showed clear associations with demographic factors (Fig. 2) [21–31]. Male participants reported higher scores (0.79) than female participants (0.77). A gradual decline in HINT-8 scores was observed with increasing age. Higher education levels were associated with better HINT-8 scores, with a gradient from elementary education to college-level attainment. Employed individuals and those with higher household incomes reported better HRQoL scores than their unemployed or lower-income counterparts.

### Ceiling effects of HINT-8

The pooled overall ceiling effects of HINT-8 (5.4% [95%CI: 2.3–8.6]) were substantially lower than those of EQ-5D (41.0% [25.4–56.6]), with both estimates derived from five studies (test for differences between instruments: χ²=19.27, *p*<0.0001) [14, 32–35] (Supplementary Material 6). Across all domains, the pooled ceiling effects from seven studies [24, 27, 32–36] were generally lower (24.3%–51.2%) than those reported in the development study (19.0%−72.0%) [14], with the exceptions of *depression* and *happiness* (Table 1). In the development study, five of eight domains had ceiling effects beyond 60% [14], whereas the pooled ceiling effects were lower than 45% except for *depression*, which exhibited the highest ceiling effect (51.2%). *Happiness* had the lowest pooled ceiling effect (24.3%), but was slightly greater than that in the development study (19.0%) [14].

**Table 1:** Pooled domain-level ceiling effects of HINT-8 from seven published studies compared with the development study

HINT-8 domain	Pooled ceiling effects [95% CI] (%)	I^2^ (%)	τ^2^	^a^Ceiling effects from the development study (%)
*Climbing stairs*	44.0 [38.8–49.2]	96.1	0.0045	**72.0**
*Pain*	35.5 [32.4–38.7]	83.5	0.0014	68.3
*Vitality*	31.3 [28.4–34.2]	79.3	0.0012	41.7
*Working*	43.4 [41.2–45.5]	76.0	0.0005	69.0
*Depression*	**51.2 [45.1**–**57.3]**	93.0	0.0065	39.7
*Memory*	34.9 [31.7–38.2]	85.1	0.0016	62.7
*Sleep*	41.5 [38.4–44.6]	86.0	0.0014	64.7
*Happiness*	24.3 [22.3–26.3]	69.3	0.0005	19.0

Note: Random-effects models were used for all pooled estimates; all heterogeneity tests were significant (Q-test *p*<0.005)

Pooled estimates were derived from Chae (2024) [24]; Kim & Kim (2022) [32]; Kim & Kim (2022) [33]; Kim et al. (2021) [34]; Kim et al. (2022) [35]; Lee (2024) [36]; and Seo et al. (2024) [27]. ^a^Values from Jo (2014) [14] are included for comparison

### Validity and reliability of the HINT-8

**Fig. 3:**
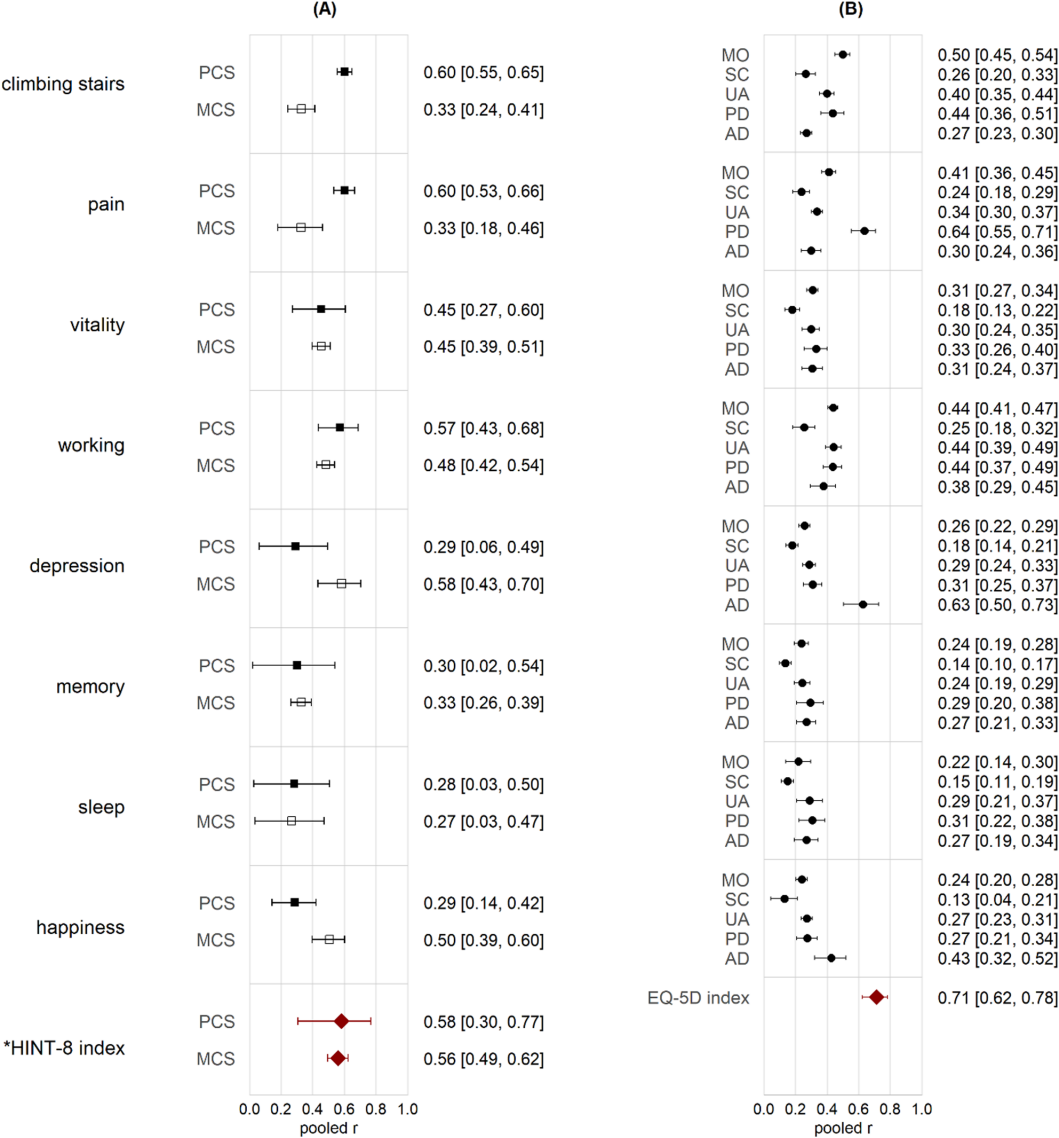
Pooled correlation coefficients between the HINT-8 and external measures. (**A**) Correlations with the physical component summary and mental component summary scores of the SF-36 from three studies. (**B**) Correlations with each domain and the index score of the EQ-5D-5L or EQ-5D-3L from six studies. Note. Random-effects models were used for all pooled estimates; Spearman correlation coefficients were used for: (1) each HINT-8 domain and the PCS/MCS of SF-36; (2) HINT-8 index and the PCS/MCS of SF-36; and (3) each HINT-8 domain and each EQ-5D domain. Pearson correlation coefficients were used to assess the association between the HINT-8 index and EQ-5D index score. ^*^Jo’s study (2014) [14] was excluded from correlation analyses of index scores because total raw scores (range: 8 − 32) were used. Only Kim & Kim (2022) [32] used the EQ-5D-3L; all of other studies used the EQ-5D-5L. PCS=physical component summary; MCS=mental component summary; MO=mobility; SC=self-care; UA=usual activity; PD=pain/discomfort; AD=anxiety/depression; pooled r=pooled correlation coefficients

HINT-8 index values showed similar correlations with the PCS (pooled *r* = 0.58) and MCS (pooled *r* = 0.56) based on two studies [35]^1^, while pooled correlations of individual HINT-8 domains were synthesized from three studies [14, 35]^1^ (Fig. 3A, Supplementary Material 7). Physical domains such as *climbing stairs* and *pain* showed higher correlations with PCS (pooled *r* = 0.60) than MCS (pooled *r* = 0.33), whereas mental health domains including *depression* and *happiness* exhibited higher correlations with MCS (pooled *r* = 0.58 and 0.50, respectively) than PCS (both *r* = 0.29). The *working* domain aligned more with PCS, and other domains (*vitality*,* memory*,* sleep*) demonstrated balanced correlations with both summary scores.

Pooled HINT-8 index values showed strong correlation with EQ-5D index (pooled *r* = 0.71) [32, 34, 35, 37]^1^, and domain-specific correlations were derived from six studies [14, 32, 34, 35, 37]^1^ (Fig. 3B, Supplementary Material 8). Expected convergent validity patterns were observed: *climbing stairs* correlated higher with EQ-5D’s *mobility* (pooled *r* = 0.50), *pain* with *pain/discomfort* (pooled *r* = 0.64), and *depression* with *anxiety/depression* (pooled *r* = 0.63). EQ-5D’s *self-care* domain showed consistently lower correlations with all HINT-8 domains (*r* = 0.13–0.26). The HINT-8 index demonstrated acceptable reproducibility, with reported ICC values of 0.690 and 0.800 from two studies [34, 35]. These findings are presented descriptively due to insufficient reporting of ICC model specifications. In contrast, the pooled kappa estimates showed moderate agreement across most domains, except for *vitality* and *happiness* (Table 2).

**Table 2:** Pooled test-retest reliability (kappa values) of the HINT-8 from two studies

Domain	Pooled Cohen’s kappa (95% CI)	I^2^ (%)	τ^2^	*p*	Pooled weighted kappa	I^2^ (%)	τ^2^	*p*
Climbing stairs	0.452 [0.345 − 0.559]	0.0	0	0.359	0.527 [0.426; 0.628]	0.0	0	0.323
Pain	0.464 [0.359 − 0.569]	0.0	0	0.540	0.502 [0.403; 0.601]	0.0	0	0.535
Vitality	0.237 [0.038 − 0.436]	75.0	0.015	0.045	0.344 [0.157; 0.530]	72.5	0.013	0.056
Working	0.479 [0.377 − 0.581]	0.0	0	0.356	0.502 [0.380; 0.625]	38.6	0.003	0.202
Depression	0.521 [0.360 − 0.683]	57.3	0.008	0.126	0.572 [0.454; 0.691]	29.0	0.002	0.235
Memory	0.420 [0.300 − 0.541]	0.0	0	0.948	0.476 [0.360; 0.592]	0.0	0	0.853
Sleep	0.402 [0.293 − 0.511]	0.0	0	0.795	0.482 [0.381; 0.584]	0.0	0	0.751
Happiness	0.326 [0.207 − 0.446]	26.9	0.002	0.242	0.391 [0.287; 0.494]	0.0	0	0.334

Note. Random-effects models were used for all pooled estimates; Pooled estimates were derived from Kim et al. (2021) [34] and Kim et al. (2022) [35], both of which included a sample size of 100 participants. Heterogeneity was assessed using I^2^ and τ^2^ statistics, and p-values correspond to Cochran’s Q test

### HINT-8 values of specific diseases

**Fig. 4:**
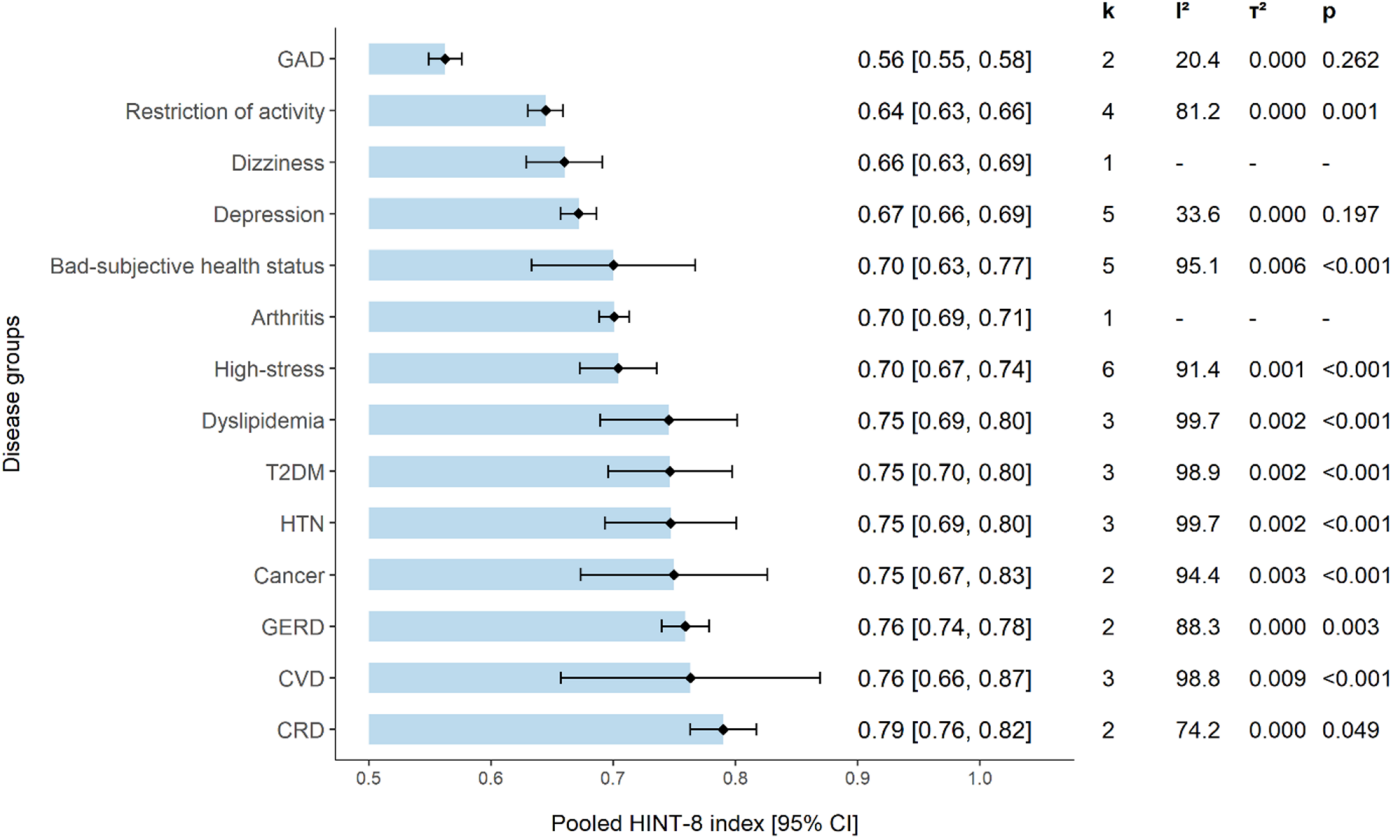
Pooled HINT-8 index values across disease groups and health statuses from sixteen studies. Note. Random-effects models were used for all pooled estimates; studies with a standard deviation of 0 were excluded from pooling; k = number of studies included. Abbreviations: GAD=generalized anxiety disorder; T2DM=diabetes mellitus; HTN=hypertension; GERD=gastroesophageal reflux disease; CVD= chronic cardiovascular disease: includes myocardial infarction, angina pectoris, and heart failure; CRD= chronic respiratory disease: includes asthma, chronic obstructive pulmonary disease, bronchiectasis, and restrictive lung disease

Pooled HINT-8 index values across various health statuses were derived from 16 studies [21–24, 27, 28, 30–33, 36, 38–42] (Fig. 4). The highest scores were observed in patients with chronic respiratory disease (pooled index: 0.79 [95% CI: 0.76–0.82]), followed by those with chronic cardiovascular disease (0.76 [0.66–0.87]) and GERD (0.76 [0.74–0.78]). The lowest score was found in patients with GAD (0.56 [0.55–0.58]). While HINT-8 scores were slightly lower than EQ-5D in some conditions (depression, arthritis, dyslipidemia, T2DM, hypertension, chronic cardiovascular disease, and respiratory disease) [32, 33, 38, 43–48], values for cancer [49] and GERD [41, 50] fell within the corresponding EQ-5D ranges (Supplementary Material 9).

## Discussion

The HINT-8 demonstrated expected construct validity, showing meaningful correlations with established generic HRQoL measures. Its pooled correlations with SF-36 (*r* = 0.58 for PCS; 0.56 for MCS) were slightly higher than those previously reported for the EQ-5D-5L in Korean samples (*r* = 0.575 − 0.59 for PCS; 0.41 − 0.553 for MCS) [14, 51], particularly for MCS. The stronger correlation with EQ-5D index compared with SF-36 components further support the validity of index-based scoring approach.

At the domain level, additional patterns help clarify the distinctive structure of HINT-8. The comparable associations of *vitality* domain with both PCS and MCS reflect its integrative nature, consistent with previous research showing that *vitality* relates broadly to overall functioning, stress, depressive symptoms, self-rated health status, and physical activity [52, 53]. This aligns with observations that SF-36 vitality domain conceptually overlaps with multiple domains rather than functioning as a discrete construct [54]. The relatively low correlation of *memory* and *sleep* domains with EQ-5D and SF-36 indicates the absence of comparable domains in those instruments, highlighting the unique construct coverage of HINT-8. This supports arguments that additional domains such as cognitive function or sleep could improve EQ-5D comprehensiveness [55–58]. The lower correlations of *working* and *happiness* domains with EQ-5D compared with SF-36 highlight the well-known structural limitation of EQ-5D, which does not include social and positive health domains. Although categorized within ‘social health’, *working* showed closer associations with PCS, suggesting that the concept may require clearer specification within the Korean context.

The substantially lower ceiling effect of HINT-8 compared with EQ-5D can be explained by its enriched domains and expanded response range, similar to improvements observed in EQ-5D-5L relative to EQ-5D-3L [11, 51, 59]. The inclusion of positive domains such as *happiness* and *vitality* may also contribute to the lower ceiling effect of HINT-8, whereas the absence of such domains in EQ-5D has been cited as a reason for its higher ceiling effects compared with SF-6D [60]. Using a commonly recommended cutoff of < 15% [61], the ceiling effects of HINT-8 falls within acceptable ranges.

Across HINT-8 domains, correlations with EQ-5D *self-care* domain were the lowest, likely due to the consistently high ceiling effect reported for this domain in previous studies [11, 49, 51, 58, 62]. High ceiling effects reduce variability and consequently attenuate correlations with other domains. Although ceiling effects may indicate limited sensitivity, they also arise when measuring healthy populations [11]. The pooled ceiling effects of most HINT-8 domains were substantially lower than those reported in the development study, reflecting its application to more diverse populations rather than the generally healthy development sample. Likewise, EQ-5D showed decreased ceiling effects when considering older age groups and increasing comorbidities [62].

However, *depression* and *happiness* domains showed higher ceiling effects. For *depression*, the single concept may not represent broader range of mental health status across various population groups. Similar results have been reported for the EQ-5D *anxiety/depression* domain, which has limited ability to capture mental health variations beyond anxiety or depression [62]. Elevated ceiling effects in the *happiness* domain may reflect the subjective nature of psychological well-being assessment. Unlike physical health domains, mental health items are influenced by psychological adaptation and response shift, where individuals adjust internal standards over time [63]. Additionally, social desirability bias may lead to more positive mental state reports regardless of objective health conditions [64].

These factors suggest subjective well-being domains may be less sensitive to population characteristic variations than physical health measures, and present unique measurement challenges. Despite showing increased ceiling effects relative to the development study, *happiness* domain still yielded the lowest scores among all domains. This highlights the complexity of capturing subjective well-being through patient-reported outcomes, which are inherently susceptible to reporting bias [65]. Its limited test-retest reliability along with *vitality* suggests that happiness-related items are particularly sensitive to time-dependent factors, such as current mood and recent psychological experiences [65]. The one-week recall period may contribute to this instability, as subjective well-being can fluctuate over short timeframes.

Given its conceptual ambiguity, happiness measurement warrants careful consideration, as it represents a “life-as-a-whole” construct, distinct from satisfactions in specific aspects of life [66]. The HINT-8 development study was grounded in positive well-being theory, which suggests that individuals can experience satisfaction with health and life despite the presence of illness, based on their subjective responses to life experiences [14]. However, the overlap in terminology between “happiness,” “well-being,” “life satisfaction,” and “quality of life” creates conceptual confusion that may compromise measurement validity and appropriate interpretation [67, 68]. Previous confirmatory factor analysis revealed that HINT-8 operates as a unidimensional construct rather than following the intended eight-domain structure (CFI = 0.952; SRMR = 0.038; RMSEA = 0.071) [52]. This finding suggests that subjective domains, particularly *happiness* and *vitality*, may not function as distinct measurement dimensions. Notably, the measurement model maintained adequate fit even when *happiness* domain was excluded [52], raising questions about its unique contribution to the overall assessment. This is consistent with the conceptual nature of happiness, which reflects a higher-order appraisal of life rather than a domain-specific aspect of health. This broader interpretation, combined with the instrument’s one-week recall period, may lead to conceptual mismatch and contribute to the item’s distinctive psychometric behavior. Therefore, it is important to re-examine whether the happiness adequately reflects its intended theoretical foundation. Future research should examine whether *happiness* domain requires subdivision into multiple sub-constructs that can be meaningfully assessed as distinct dimensions, or whether alternative approaches to measuring subjective well-being would better serve the instrument’s intended purpose. Despite these domain-level concerns, the predominance of a general HRQoL factor supports the psychometric appropriateness of using a single summary index score, particularly given that HINT-8 is designed as a preference-based instrument for clinical and health-economic applications.

Beyond psychometric performance, HINT-8 showed expected differences across sociodemographic groups, reflecting anticipated gradients in health status. Pooled HINT-8 index across different disease populations also revealed distinctive patterns of HRQoL impairment. Populations with mental disorders or psychological distress demonstrated lower HINT-8 scores than those with other chronic diseases. In contrast, EQ-5D index scores from independent studies showed relatively consistent values across various disease groups. This implies that HINT-8 may be potentially sensitive to the burden of mental health conditions and may exhibit varying discriminative ability across disease groups. This interpretation is further supported by prior evidence, with HINT-8 index scores demonstrating moderate to strong correlations with established mental health measures, such as the GAD 7-Item Scale (ranging from 0.42 to 0.72 across studies [21, 39, 42]), and self-rated health status (0.50 − 0.58 [21, 39]). Further investigation is warranted in populations with severe health conditions and diverse psychiatric disorders to better establish its sensitivity and generalizability.

To our knowledge, this is the first study to comprehensively review the psychometric properties of HINT-8 through evidence synthesis. Our review highlights the distinctive contribution of the HINT-8 as a generic HRQoL instrument. Through the inclusion of domains such as vitality and social functioning, which are not covered in EQ-5D, HINT-8 provides broader conceptual coverage and improved sensitivity across diverse populations and health conditions. Furthermore, as a preference-based measure that yields a single index value, HINT-8 can be directly applied in cost-utility analyses. This facilitates comparability with other established instruments and supports international benchmarking while capturing broader aspects of quality of life. However, over half (58%) of the identified studies relied on unweighted domain scores, raising concerns about underutilization of the instrument’s full potential. Future applications should incorporate preference-weighted index to enhance the accuracy and policy relevance of HINT-8 assessments.

A few methodological limitations should be acknowledged. First, reliance on KNHANES data may have introduced systematic bias and limited control for unmeasured confounders. Second, the limited number of studies reporting HINT-8 psychometric properties necessitated the inclusion of overlapping datasets, potentially inflating the precision of pooled estimates and reducing evidence diversity. Third, our synthetic approach was constrained by considerable heterogeneity among the included studies. Differences in the study populations and designs may have affected the consistency and interpretation of the results. Particularly during the synthesis of HINT-8 index scores by disease group, pooling was conducted using overarching disease labels to ensure consistency. This approach did not account for differences in disease severity or comorbid conditions, as many studies lacked detailed diagnostic information. Although we applied a random-effects framework with weighted values, residual heterogeneity could not be completely ruled out. The random-effects model may also have produced unstable estimates of between-study variance because the number of studies included in the validation analyses was small.

Finally, the exclusive inclusion of studies conducted in Korea limits the generalizability of these findings to non-Korean populations and different healthcare contexts. Nevertheless, within the contest of the considerable heterogeneity noted above, the findings support the applicability of HINT-8 as a generic measure across diverse population groups, reflecting its robust methodological foundation and psychometric performance.

## Conclusions

HINT-8 maintains sensitivity and validity across heterogeneous populations based on the studies examined, supporting its utility as a robust generic measure.

## Supplementary Information

Below is the link to the electronic supplementary material.

Supplementary Material 1

Supplementary Material 2

Supplementary Material 3

Supplementary Material 4

Supplementary Material 5

Supplementary Material 6

Supplementary Material 7

Supplementary Material 8

Supplementary Material 9

## Data Availability

The data analyzed in this study are publicly available and reported in published papers, which are cited in the reference list.

## Footnotes

1 Additional unpublished data from our research group support these findings (manuscript under review).

## Abbreviations

HRQoL: Health-related quality of life
EQ-5D: EuroQol-5 dimension
SF-36: Short-form 36 Questionnaire
EQ-VAS: EuroQol-visual analog scale
KNHANES: Korea National Health and Nutrition Examination Survey
CI: Confidence interval
ICC: Intraclass correlation coefficient
COSMIN: COnsensus-based Standards for the Selection of health Measurement INstruments
GERD: Gastroesophageal reflux disease
GAD: Generalized anxiety disorder
T2DM: Type 2 diabetes mellitus
HTN: Hypertension
