# Supplementary material for: Psychometric properties of the health-related quality of life instrument with 8 items: a systematic review and meta-analysis
Source: Health Qual Life Outcomes. 2026 Mar 4;24:47. doi: 10.1186/s12955-026-02494-z (PMC13067613; doi:10.1186/s12955-026-02494-z)
Supplement: Supplementary file 1 — Supplementary Material 1 [file 12955_2026_2494_MOESM1_ESM.pdf]

Supplementary Material 1. Detailed search strategies for identifying studies using the HINT-8 instrument across databases

Database	Search string	Search date
PubMed	"HINT-8" OR "HINT eight" OR ("health-related quality of life" AND instrument AND ("8 items" OR "8 item" OR "eight items" OR "eight item"))	2025-03-24 (revised on 2025-11-12)
KoreaMed	((HINT-8[ALL] OR HINT-eight[ALL]) OR Health-related quality of life instrument with 8 items[ALL])	
KMbase	Advanced search was used with the following keywords combined with OR: HINT-8; HINT-eight; Health-related quality of life instrument with 8 items.<br>The corresponding search syntax: ((HINT-8|title) OR (HINT-eight|title)) OR (Health-related quality of life instrument with 8 items|title)	
KISS	"HINT-8" OR "HINT-eight" OR "Health-related quality of life instrument with 8 items" in advanced research	
Google Scholar	"HINT-8" OR "HINT-eight" OR "Health-related quality of life instrument with 8 items"	2025-03-25 (revised on 2025-11-12)
