# Supplementary material for: Psychometric properties of the health-related quality of life instrument with 8 items: a systematic review and meta-analysis
Source: Health Qual Life Outcomes. 2026 Mar 4;24:47. doi: 10.1186/s12955-026-02494-z (PMC13067613; doi:10.1186/s12955-026-02494-z)
Supplement: Supplementary file 2 — Supplementary Material 2 [file 12955_2026_2494_MOESM2_ESM.pdf]

Supplementary Material 2. Characteristics of included studies

First author (year)	Study design	Population	Age (mean ± SD)	Sample (n)	Study setting	Instruments used	Exposures/ other outcomes	HINT-8 scoring	
Jo (2014)	Validation study	General adults	Age-stratified	300	Community-based survey	SF-36, EQ-5D-5L, EQ-5D-3L, EQ-VAS	Instrument development	Item score summation	[14]
Kim et al. (2021)	Validation study	Breast cancer patients (≥30yrs)	54.4±9.1	300	Tertiary hospital	EQ-5D-5L, EQ-VAS, FACT-B	Validity, reliability	Index	[34]
Choi et al. (2024)	Validation study	Family caregivers of people with dementia	67.02±11.49	47	Public dementia centers and long-term care facilities	EQ-5D-5L, SZBI	Validity	Index	[37]
Kim & Kim (2022)	Validation study	Older adults (≥65yrs)	Age-stratified	1,519	2019 KNHANES	EQ-5D-3L	Validity	Index	[32]
Kim et al. (2022)	Validation study	Type 2 diabetes mellitus patients	60.4±11.6	300	Tertiary hospital	EQ-5D-5L, EQ-VAS, SF-36, SF-6D	Validity, reliability	Index	[35]
Chung et al. (2024)	Prospective single-arm study	Patients with chronic respiratory and cardiovascular diseases	67.0 [60.0–70.8][a]	75	Tertiary hospital	EQ-5D-5L	Application-based rehabilitation/ clinical indicators	Index	[38]
Kim et al. (2022)	Cross-sectional	Patients with cancer, cancer survivors, and general populations	30-69	3,085	2019 KNHANES	-	Employment status	Index	[28]
Lee (2024)	Cross-sectional	Older adults (≥65yrs)	Age-stratified	1,455	2019 KNHANES	-	HGS, chronic diseases, mental health, nutrition intake	Index	[26]
Park (2023)	Cross-sectional	Older adults with arthritis (≥65yrs)	Age-stratified	423	2021 KNHANES	GAD-7	-	Index	[39]
Lee & Jun (2023)	Cross-sectional	Young breast cancer survivors undergoing endocrine therapy	42.75 ± 6.60	133	Online patient community	MRS, MSPSS, CD-RISC	Menopausal symptoms, social support, resilience	Index	[40]
Kim & Kim (2022)	Cross-sectional	Patients with chronic obstructive pulmonary disease (≥40yrs)	Age-stratified	451	2019 KNHANES	EQ-5D-3L	-	Index	[33]
Lee (2024)	Cross-sectional	Patients with dyslipidemia	Age-stratified	1,926	2019, 2021 KNHANES	-	-	Index	[36]
Park & Lee (2024)	Cross-sectional	Middle-aged and older adults	40-64	2,380	2021 KNHANES	GAD-7	Disease morbidity	Index	[21]
Heo & Jang (2023)	Cross-sectional	Adults in their 20s	19-29	750	2019 KNHANES	-	-	Index	[22]

Park et al. (2019)	Multicenter prospective study	Patients with GERD	53.3	51	Tertiary hospital	GERD-HRQL, EQ-5D (version not specified), EQ-VAS	Anti-reflux surgery	Index	[41]
Seo et al. (2024)	Cross-sectional	Adults with or without dizziness/imbalance (≥40yrs)	Age-stratified	3,959	2021 KNHANES	GAD-7	-	Index	[27]
Lee (2024)	Cross-sectional	Older adults (≥65yrs)	72.99±0.197	1,415	2021 KNHANES	GAD-7	-	Index	[31]
Jang (2024)	Cross-sectional	Middle-aged widowed women	57.20±5.83	55	2021 KNHANES	-	-	Index	[30]
Jung & An (2024)	Cross-sectional	One-person households	20-64	839	2019, 2021 KNHANES	-	-	Index	[23]
Chae (2024)	Cross-sectional	One-person households	young (19-39), middle-aged (40-64), older (≥65)	958	2021 KNHANES	-	-	Index	[24]
Kim et al. (2022)	Cross-sectional	Healthy adults (50-80 yrs)	60.2±6.7	153	Tertiary hospital	-	Musculoskeletal health	Index	[29]
Kim & Kang (2024)	Cross-sectional	Patients with gastroesophageal reflux disease	Age-stratified	143	Online patient community	FSSG, GAD-7, HPLP II	-	Index	[42]
Lee et al. (2023)	Cross-sectional	Young adults (19–34yrs) living alone	29.43±3.24	229	Online community for single-person households	PHQ-9, Resilience Questionnaire-53	-	Index	[25]

Note: All included studies were conducted in Korean populations. [a]Age was reported in median and interquartile range.

Abbreviations: FACT-B= Functional Assessment of Cancer Therapy; SZBI= Short Zarit Burden Interview; HGS= Handgrip strength; GAD-7= Generalized anxiety disorder scale-7; MRS= Menopause rating scale; MSPSS= Multidimensional scale of perceived social support; CD-RISC= Connor-Davidson resilience scale; GERD= Gastroesophageal reflux disease; FSSG= Frequency scale for the symptoms of gastroesophageal reflux disease; HPLP II= Health Promoting Lifestyle Profile II; PHQ-9= Patient health questionnaire-9
