# Supplementary material for: Psychometric properties of the health-related quality of life instrument with 8 items: a systematic review and meta-analysis
Source: Health Qual Life Outcomes. 2026 Mar 4;24:47. doi: 10.1186/s12955-026-02494-z (PMC13067613; doi:10.1186/s12955-026-02494-z)
Supplement: Supplementary file 3 — Supplementary Material 3 [file 12955_2026_2494_MOESM3_ESM.pdf]

Supplementary Material 3. Study inclusion and exclusion status for each pooled analysis

Pooled outcome	Subgroups	Included studies for each pooling category	Studies excluded due to overlapping datasets
Demographic factors	Male, Female	Heo & Jang (2023) [22]; Jung & An (2024) [23]; Kim et al. (2022) [29]; Kim et al. (2022) [28]; Lee (2024) [26]; Lee et al. (2023) [25]; Lee (2024) [31]; Park & Lee (2024) [21]; Seo et al. (2024) [27]	Chae (2024) [24]
	20-29	Heo & Jang (2023) [22]; Jung & An (2024) [23]; Lee et al. (2023) [25]	
	30-39	Jung & An (2024) [23]; Kim et al. (2022) [28]; Lee et al. (2023) [25]	
	40-49, 50-59	Jung & An (2024) [23]; Kim et al. (2022) [28]; Seo et al. (2024) [27]	
	over 60	Jung & An (2024) [23]; Kim et al. (2022) [28]; Lee (2024) [26]; Lee (2024) [31]; Seo et al. (2024) [27]	Chae (2024) [24]
	Elementary	Jang (2024) [30]; Lee (2024) [26]; Lee (2024) [31]; Park & Lee (2024) [21]; Seo et al. (2024) [27]	
	Middle school	Chae (2024) [24]; Jang (2024) [30]; Lee (2024) [26]; Lee (2024) [31]; Park & Lee (2024) [21]; Seo et al. (2024) [27]	
	High school, College	Heo & Jang (2023) [22]; Jang (2024) [30]; Jung & An (2024) [23]; Kim et al. (2022) [28]; Lee (2024) [26]; Lee et al. (2023) [25]; Lee (2024) [31]; Park & Lee (2024) [21]; Seo et al. (2024) [27]	Chae (2024) [24]
	Not employed, Employed	Chae (2024) [24]; Kim et al. (2022) [28]; Park & Lee (2024) [21]	
	Low/Middle/ High income	Jang (2024) [30]; Jung & An (2024) [23]; Kim et al. (2022) [28]; Lee (2024) [26]; Lee et al. (2023) [25]; Lee (2024) [31]; Park & Lee (2024) [21]; Seo et al. (2024) [27]	Chae (2024) [24]
Ceiling effects	Overall ceiling effect	Jo (2014) [14]; Kim & Kim (2022) [32]; Kim & Kim (2022) [33]; Kim et al. (2021) [34]; Kim et al. (2022) [35]	
	Domain-level ceiling effect	Chae (2024) [24]; Kim & Kim (2022) [32]; Kim & Kim (2022) [33]; Kim et al. (2021) [34]; Kim et al. (2022) [35]; Lee (2024) [36]; Seo et al. (2024) [27]	Lee (2024) [26]
[a]Construct validity (correlation coefficients)	HINT-8/SF-36	Jo (2014) [14]; Kim et al. (2022) [35]	
	HINT-8/EQ-5D	Choi et al. (2024) [37]; Jo (2014) [14]; Kim & Kim (2022) [32]; Kim et al. (2021) [34]; Kim et al. (2022) [35]	
Reliability	Kappa, ICC	Kim et al. (2021) [34]; Kim et al. (2022) [35]	
Disease subgroups	GAD	Lee (2024) [31]; Seo et al. (2024) [27]	
	Restriction of activity	Chae (2024) [24]; Park (2023) [39]; Park & Lee (2024) [21]; Seo et al. (2024) [27]	

Disease subgroups (cont.)	Dizziness	Seo et al. (2024) [27]	
	Depression	Chae (2024) [24]; Heo & Jang (2023) [22]; Jang (2024) [30]; Kim & Kim (2022) [32]; Park & Lee (2024) [21]	
	High-stress	Heo & Jang (2023) [22]; Jang (2024) [30]; Jung & An (2024) [23]; Kim & Kim (2022) [32]; Kim & Kim (2022) [33]; Seo et al. (2024) [27]	Chae (2024) [24]
	Bad-subjective health status	Chae (2024) [24]; Heo & Jang (2023) [22]; Kim & Kim (2022) [33]; Lee (2024) [31]; Seo et al. (2024) [27]	
	Arthritis	Park (2023) [39]	
	Dyslipidemia	Kim et al. (2022) [28]; Lee (2024) [36]; Park & Lee (2024) [21]; Seo et al. (2024) [27]	
	T2DM, HTN	Kim et al. (2022) [28]; Park & Lee (2024) [21]; Seo et al. (2024) [27]	
	Cancer	Kim et al. (2022) [28]; Lee & Jun (2023) [40]	
	GERD	Kim & Kang (2024) [42]; Park et al. (2019) [41]	
	Chronic cardiovascular disease	Chung et al. (2024) [38]; Kim & Kim (2022) [32]; Park & Lee (2024) [21]	
	Chronic respiratory disease	Chung et al. (2024) [38]; Kim & Kim (2022) [33]	

Note: [a]Additional unpublished data from our research group was included.
