# Supplementary material for: Psychometric properties of the health-related quality of life instrument with 8 items: a systematic review and meta-analysis
Source: Health Qual Life Outcomes. 2026 Mar 4;24:47. doi: 10.1186/s12955-026-02494-z (PMC13067613; doi:10.1186/s12955-026-02494-z)
Supplement: Supplementary file 4 — Supplementary Material 4 [file 12955_2026_2494_MOESM4_ESM.pdf]

Supplementary Material 4. Risk of bias assessment of included studies using the RoBANS 2 checklist

Study	D1	D2	D3	D4	D5	D6	D7	D8		Study design
Chung et al. (2024)	Low	Low	**High**	Low	Low	Low	**Unclear**	Low	[38]	Cohort
Park et al. (2019)	Low	Low	**High**	Low	Low	Low	**High**	Low	[41]	
Kim et al. (2022)	N/A	Low	Low	Low	Low	Low	Low	Low	[28]	Cross-sectional
Lee (2024)	N/A	Low	Low	Low	Low	Low	Low	Low	[26]	
Park (2023)	N/A	Low	Low	Low	Low	Low	Low	Low	[39]	
Lee & Jun (2023)	N/A	Low	Low	Low	Low	Low	Low	Low	[40]	
Kim & Kim (2022)	N/A	Low	Low	Low	Low	Low	Low	Low	[33]	
Lee (2024)	N/A	Low	**High**	Low	Low	Low	Low	Low	[36]	
Park & Lee (2024)	N/A	Low	**High**	Low	Low	Low	Low	Low	[21]	
Heo & Jang (2023)	N/A	Low	Low	Low	Low	Low	Low	Low	[22]	
Seo et al. (2024)	N/A	Low	Low	Low	Low	Low	Low	Low	[27]	
Lee (2024)	N/A	Low	Low	Low	Low	Low	Low	Low	[31]	
Jang (2024)	N/A	Low	Low	Low	Low	Low	Low	Low	[30]	
Jung & An (2024)	N/A	Low	Low	Low	Low	Low	Low	Low	[23]	
Chae (2024)	N/A	Low	Low	Low	Low	Low	Low	Low	[24]	
Kim et al. (2022)	N/A	Low	Low	**High**	Low	Low	Low	Low	[29]	
Kim & Kang (2024)	N/A	Low	Low	Low	Low	Low	Low	Low	[42]	
Lee et al. (2023)	N/A	Low	Low	Low	Low	Low	Low	Low	[25]	

Note: RoBANS= Risk of Bias Assessment Tool for Nonrandomized Studies of Interventions; D1= Comparability of the target group; D2= Target group selection; D3= Cofounders; D4= Measurement of intervention/exposure; D5= Blinding of assessors; D6= Outcome assessment; D7=Incomplete outcome data; D8= Selective outcome reporting. Domain 1 was rated as "not applicable" for single-group cross-sectional studies.
