# Supplementary material for: Psychometric properties of the health-related quality of life instrument with 8 items: a systematic review and meta-analysis
Source: Health Qual Life Outcomes. 2026 Mar 4;24:47. doi: 10.1186/s12955-026-02494-z (PMC13067613; doi:10.1186/s12955-026-02494-z)
Supplement: Supplementary file 5 — Supplementary Material 5 [file 12955_2026_2494_MOESM5_ESM.pdf]

Supplementary Material 5. COSMIN risk of bias and criteria rating results of five studies

**Measurement property** (studies included per property)	**COSMIN risk of bias**	**COSMIN criteria ratings** (+/-/?)	**Summary of results**	**GRADE approach**
**PROM development** (n=1)	adequate	NA	NA	NA
**Content validity** (n=1)	adequate	sufficient (+)	Based on the development study:<br>- Items were relevant<br>- Minor concerns for comprehensibility in a few domains (e.g., *working*, *happiness*)	Moderate
**Reliability** (n=3)	doubtful (n=1)<br>inadequate (n=2)	sufficient (+)	- Sufficient ICC values<br>- Low certainty due to differing measurement conditions, and unreported ICC models	Low
**Hypotheses testing for construct validity** (n=5)	very good	sufficient (+)	- Consistent results meeting hypotheses	High

Note. Risk of bias: very good, adequate, doubtful, inadequate; Criteria ratings: sufficient (+), insufficient (-), indeterminate (?)
