# Supplementary material for: Psychometric properties of the health-related quality of life instrument with 8 items: a systematic review and meta-analysis
Source: Health Qual Life Outcomes. 2026 Mar 4;24:47. doi: 10.1186/s12955-026-02494-z (PMC13067613; doi:10.1186/s12955-026-02494-z)
Supplement: Supplementary file 6 — Supplementary Material 6 [file 12955_2026_2494_MOESM6_ESM.pdf]

Supplementary Material 6. Forest plots of overall ceiling effects for HINT-8 and EQ-5D (3L/5L)

Study	Events	Total	Proportion	95% CI	Weight (%)
**instrument = HINT-8**					
Jo (2014) [14]	37	300	0.123	[0.088; 0.166]	17.0
Kim & Kim (2022) [32]	52	1519	0.034	[0.026; 0.045]	22.0
Kim & Kim (2022) [33]	26	451	0.058	[0.038; 0.083]	20.2
Kim et al. (2021) [34]	7	300	0.023	[0.009; 0.047]	21.0
Kim et al. (2022) [35]	14	300	0.047	[0.026; 0.077]	19.8
**Random effects model**		**2870**	**0.054**	**[0.023; 0.085]**	**100.0**
Heterogeneity: $I^2 = 85.3\%$, $\tau^2 = 0.0011$, $p < 0.0001$					
**instrument = EQ-5D**					
Jo (2014) [14]	155	300	0.517	[0.459; 0.574]	19.8
Kim & Kim (2022) [32]	752	1519	0.495	[0.470; 0.521]	20.2
Kim & Kim (2022) [33]	270	451	0.599	[0.552; 0.644]	20.0
Kim et al. (2021) [34]	63	300	0.210	[0.165; 0.261]	20.0
Kim et al. (2022) [35]	69	300	0.230	[0.184; 0.282]	20.0
**Random effects model**		**2870**	**0.410**	**[0.254; 0.566]**	**100.0**
Heterogeneity: $I^2 = 98.4\%$, $\tau^2 = 0.0311$, $p < 0.0001$					
Test for subgroup differences: $\chi^2_1 = 19.27$, df = 1 ($p < 0.0001$)					

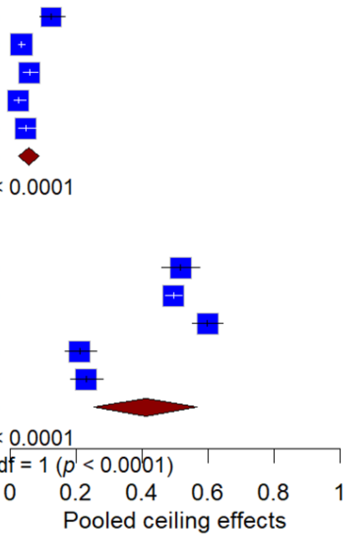

Note. The EQ-5D-3L was used in Kim & Kim (2022) [32] and Kim & Kim (2022) [33].
