# Supplementary material for: Psychometric properties of the health-related quality of life instrument with 8 items: a systematic review and meta-analysis
Source: Health Qual Life Outcomes. 2026 Mar 4;24:47. doi: 10.1186/s12955-026-02494-z (PMC13067613; doi:10.1186/s12955-026-02494-z)
Supplement: Supplementary file 7 — Supplementary Material 7 [file 12955_2026_2494_MOESM7_ESM.pdf]

Supplementary Material 7. Forest plots of correlations between HINT-8 and SF-36

Climbing stairs

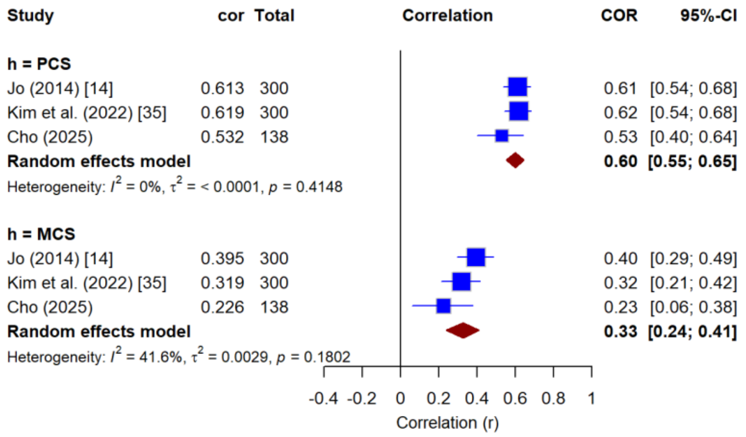

Pain

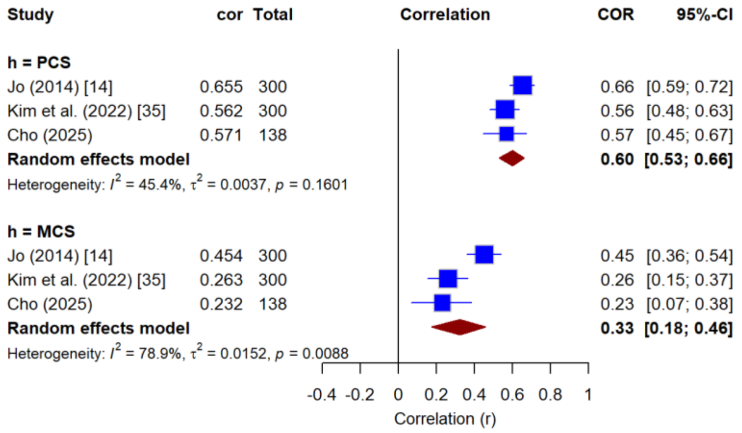

Vitality

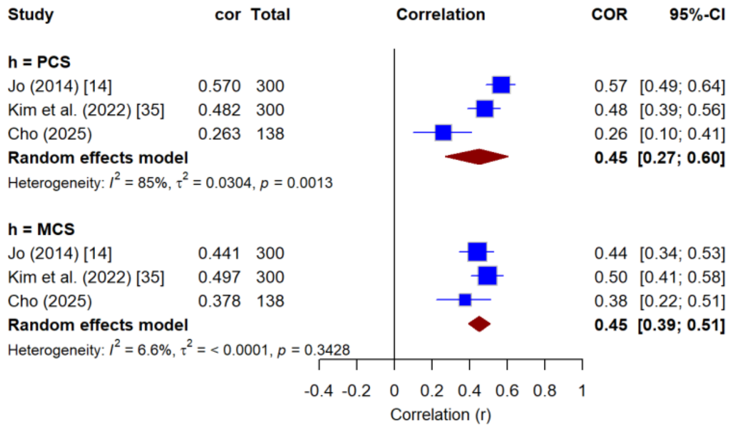

Working

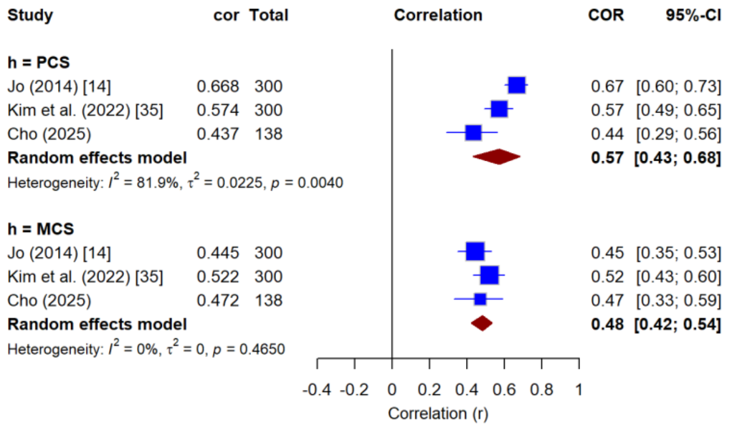

Depression

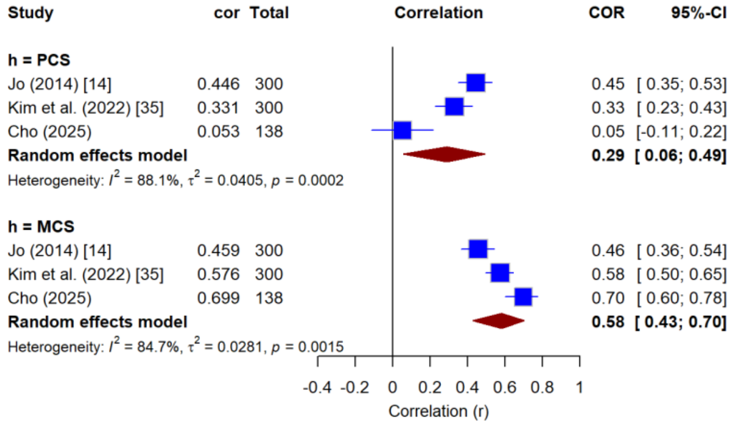

Memory

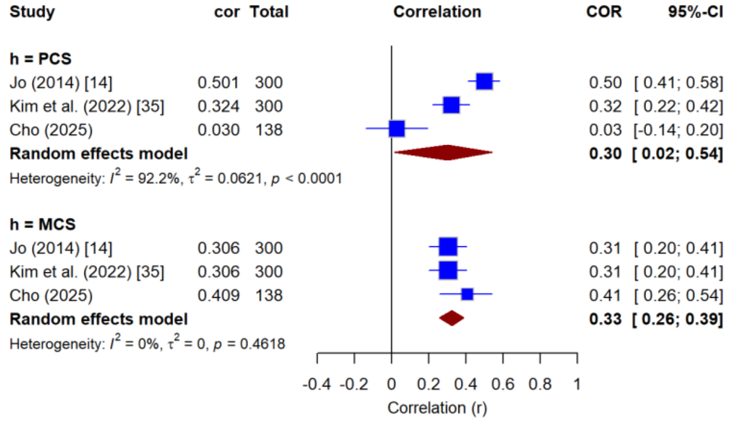

Sleep

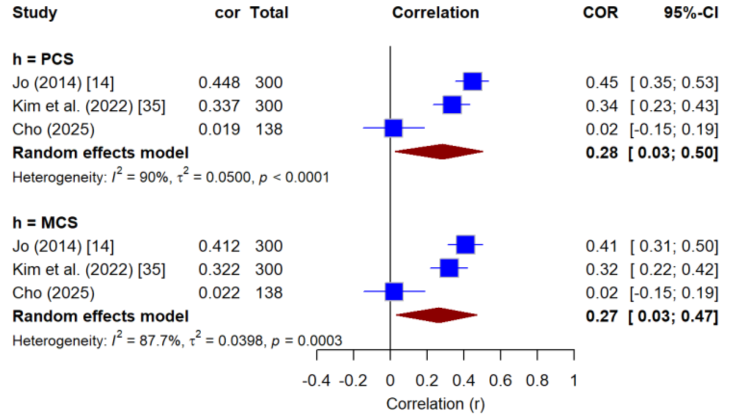

Happiness

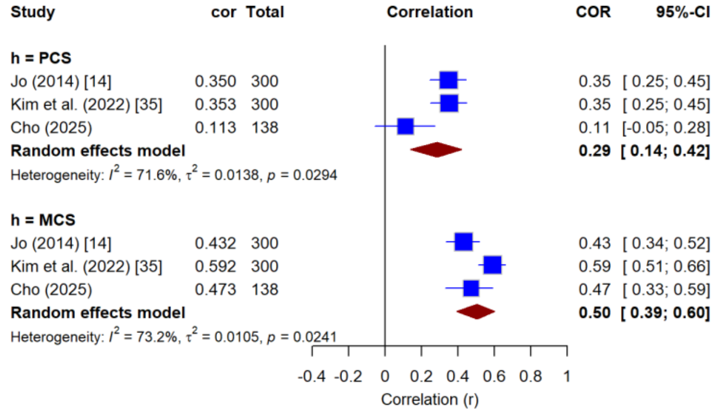

HINT-8 index

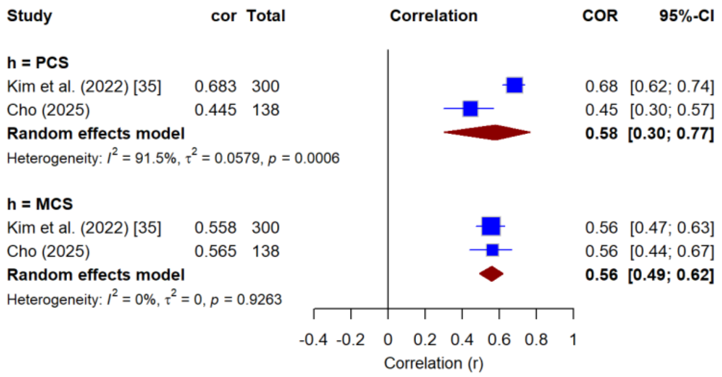

Note. Cho (2025) is additional unpublished data from our research group.
