# Supplementary material for: Psychometric properties of the health-related quality of life instrument with 8 items: a systematic review and meta-analysis
Source: Health Qual Life Outcomes. 2026 Mar 4;24:47. doi: 10.1186/s12955-026-02494-z (PMC13067613; doi:10.1186/s12955-026-02494-z)
Supplement: Supplementary file 8 — Supplementary Material 8 [file 12955_2026_2494_MOESM8_ESM.pdf]

Supplementary Material 8. Forest plots of correlations between HINT-8 and EQ-5D

Climbing stairs

Study	cor	Total	COR	95%-CI
**h = Mobility**				
Jo (2014) [14]	0.403	300	0.40	[0.30; 0.49]
Kim et al. (2021) [34]	0.493	300	0.49	[0.40; 0.57]
Kim et al. (2022) [35]	0.509	300	0.51	[0.42; 0.59]
Kim & Kim (2022) [32]	0.520	1519	0.52	[0.48; 0.56]
Choi et al. (2024) [37]	0.721	47	0.72	[0.55; 0.84]
Cho (2025)	0.461	138	0.46	[0.32; 0.58]
**Random effects model**			**0.50**	**[0.45; 0.54]**
Heterogeneity: $I^2 = 56.8\%$, $\tau^2 = 0.0023$, $p = 0.0410$				
**h = Self-care**				
Jo (2014) [14]	0.203	300	0.20	[0.09; 0.31]
Kim et al. (2021) [34]	0.165	300	0.16	[0.05; 0.27]
Kim et al. (2022) [35]	0.349	300	0.35	[0.25; 0.44]
Kim & Kim (2022) [32]	0.300	1519	0.30	[0.25; 0.35]
Choi et al. (2024) [37]	0.360	47	0.36	[0.08; 0.59]
Cho (2025)	0.239	138	0.24	[0.08; 0.39]
**Random effects model**			**0.26**	**[0.20; 0.33]**
Heterogeneity: $I^2 = 46.8\%$, $\tau^2 = 0.0032$, $p = 0.0939$				
**h = Usual activities**				
Jo (2014) [14]	0.378	300	0.38	[0.28; 0.47]
Kim et al. (2021) [34]	0.321	300	0.32	[0.22; 0.42]
Kim et al. (2022) [35]	0.366	300	0.37	[0.26; 0.46]
Kim & Kim (2022) [32]	0.430	1519	0.43	[0.39; 0.47]
Choi et al. (2024) [37]	0.610	47	0.61	[0.39; 0.76]
Cho (2025)	0.411	138	0.41	[0.26; 0.54]
**Random effects model**			**0.40**	**[0.35; 0.44]**
Heterogeneity: $I^2 = 41.3\%$, $\tau^2 = 0.0012$, $p = 0.1298$				
**h = Pain/Discomfort**				
Jo (2014) [14]	0.556	300	0.56	[0.47; 0.63]
Kim et al. (2021) [34]	0.365	300	0.36	[0.26; 0.46]
Kim et al. (2022) [35]	0.412	300	0.41	[0.31; 0.50]
Kim & Kim (2022) [32]	0.380	1519	0.38	[0.34; 0.42]
Choi et al. (2024) [37]	0.601	47	0.60	[0.38; 0.76]
Cho (2025)	0.389	138	0.39	[0.24; 0.52]
**Random effects model**			**0.44**	**[0.36; 0.51]**
Heterogeneity: $I^2 = 70\%$, $\tau^2 = 0.0080$, $p = 0.0052$				
**h = Anxiety/Depression**				
Jo (2014) [14]	0.336	300	0.34	[0.23; 0.43]
Kim et al. (2021) [34]	0.241	300	0.24	[0.13; 0.34]
Kim et al. (2022) [35]	0.279	300	0.28	[0.17; 0.38]
Kim & Kim (2022) [32]	0.260	1519	0.26	[0.21; 0.31]
Choi et al. (2024) [37]	0.383	47	0.38	[0.11; 0.60]
Cho (2025)	0.215	138	0.21	[0.05; 0.37]
**Random effects model**			**0.27**	**[0.23; 0.30]**
Heterogeneity: $I^2 = 0\%$, $\tau^2 = 0$, $p = 0.6650$				

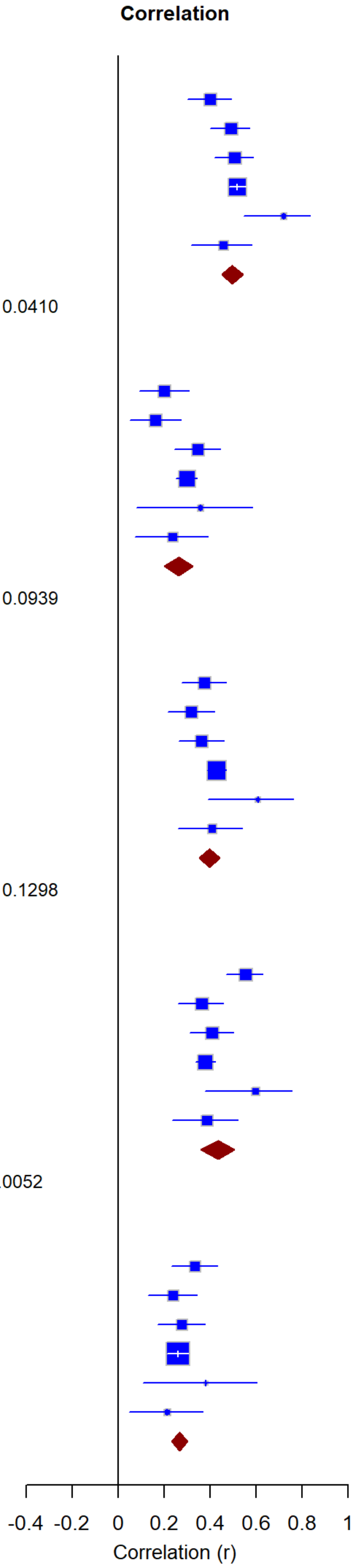

Pain

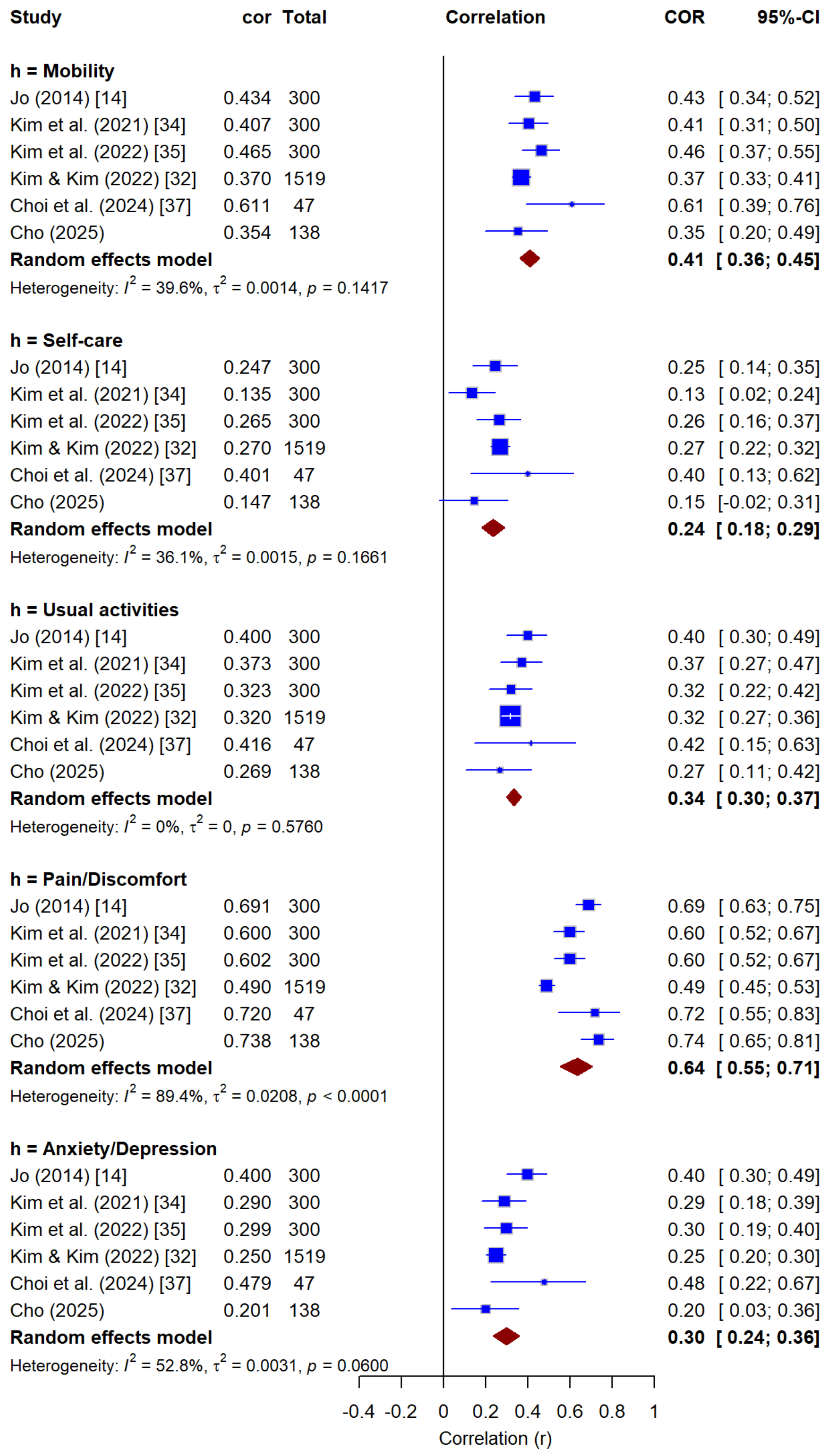
Study cor Total Correlation COR 95%-CI
h = Mobility
Jo (2014) [14] 0.434 300 0.43 [0.34; 0.52]
Kim et al. (2021) [34] 0.407 300 0.41 [0.31; 0.50]
Kim et al. (2022) [35] 0.465 300 0.46 [0.37; 0.55]
Kim & Kim (2022) [32] 0.370 1519 0.37 [0.33; 0.41]
Choi et al. (2024) [37] 0.611 47 0.61 [0.39; 0.76]
Cho (2025) 0.354 138 0.35 [0.20; 0.49]
Random effects model 0.41 [0.36; 0.45]
Heterogeneity: I^2 = 39.6%, τ^2 = 0.0014, p = 0.1417
h = Self-care
Jo (2014) [14] 0.247 300 0.25 [0.14; 0.35]
Kim et al. (2021) [34] 0.135 300 0.13 [0.02; 0.24]
Kim et al. (2022) [35] 0.265 300 0.26 [0.16; 0.37]
Kim & Kim (2022) [32] 0.270 1519 0.27 [0.22; 0.32]
Choi et al. (2024) [37] 0.401 47 0.40 [0.13; 0.62]
Cho (2025) 0.147 138 0.15 [-0.02; 0.31]
Random effects model 0.24 [0.18; 0.29]
Heterogeneity: I^2 = 36.1%, τ^2 = 0.0015, p = 0.1661
h = Usual activities
Jo (2014) [14] 0.400 300 0.40 [0.30; 0.49]
Kim et al. (2021) [34] 0.373 300 0.37 [0.27; 0.47]
Kim et al. (2022) [35] 0.323 300 0.32 [0.22; 0.42]
Kim & Kim (2022) [32] 0.320 1519 0.32 [0.27; 0.36]
Choi et al. (2024) [37] 0.416 47 0.42 [0.15; 0.63]
Cho (2025) 0.269 138 0.27 [0.11; 0.42]
Random effects model 0.34 [0.30; 0.37]
Heterogeneity: I^2 = 0%, τ^2 = 0, p = 0.5760
h = Pain/Discomfort
Jo (2014) [14] 0.691 300 0.69 [0.63; 0.75]
Kim et al. (2021) [34] 0.600 300 0.60 [0.52; 0.67]
Kim et al. (2022) [35] 0.602 300 0.60 [0.52; 0.67]
Kim & Kim (2022) [32] 0.490 1519 0.49 [0.45; 0.53]
Choi et al. (2024) [37] 0.720 47 0.72 [0.55; 0.83]
Cho (2025) 0.738 138 0.74 [0.65; 0.81]
Random effects model 0.64 [0.55; 0.71]
Heterogeneity: I^2 = 89.4%, τ^2 = 0.0208, p < 0.0001
h = Anxiety/Depression
Jo (2014) [14] 0.400 300 0.40 [0.30; 0.49]
Kim et al. (2021) [34] 0.290 300 0.29 [0.18; 0.39]
Kim et al. (2022) [35] 0.299 300 0.30 [0.19; 0.40]
Kim & Kim (2022) [32] 0.250 1519 0.25 [0.20; 0.30]
Choi et al. (2024) [37] 0.479 47 0.48 [0.22; 0.67]
Cho (2025) 0.201 138 0.20 [0.03; 0.36]
Random effects model 0.30 [0.24; 0.36]
Heterogeneity: I^2 = 52.8%, τ^2 = 0.0031, p = 0.0600
-0.4 -0.2 0 0.2 0.4 0.6 0.8 1
Correlation (r)

Vitality

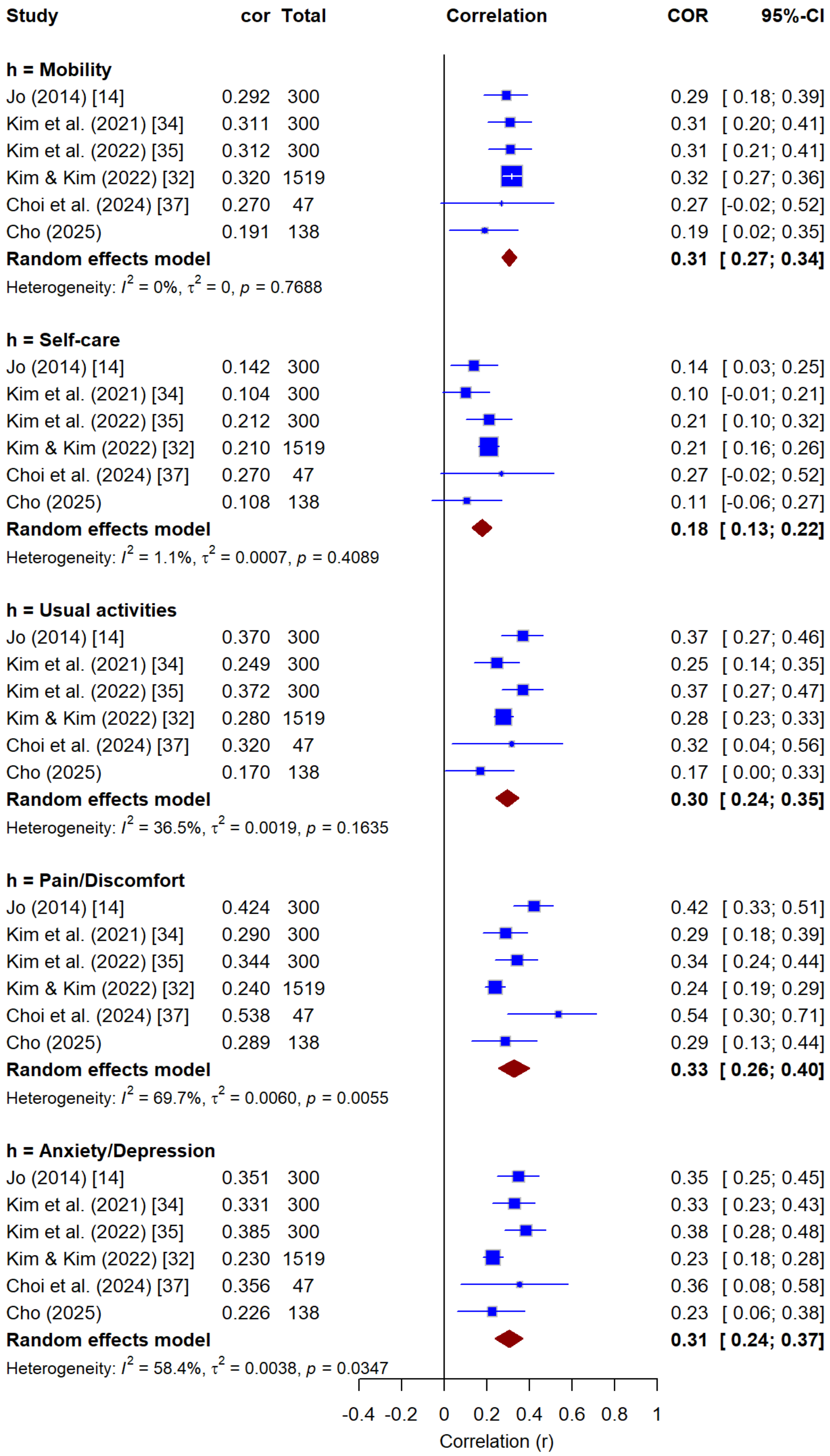
Study cor Total Correlation COR 95%-CI
h = Mobility
Jo (2014) [14] 0.292 300 0.29 [0.18; 0.39]
Kim et al. (2021) [34] 0.311 300 0.31 [0.20; 0.41]
Kim et al. (2022) [35] 0.312 300 0.31 [0.21; 0.41]
Kim & Kim (2022) [32] 0.320 1519 0.32 [0.27; 0.36]
Choi et al. (2024) [37] 0.270 47 0.27 [-0.02; 0.52]
Cho (2025) 0.191 138 0.19 [0.02; 0.35]
Random effects model 0.31 [0.27; 0.34]
Heterogeneity: I^2 = 0%, τ^2 = 0, p = 0.7688
h = Self-care
Jo (2014) [14] 0.142 300 0.14 [0.03; 0.25]
Kim et al. (2021) [34] 0.104 300 0.10 [-0.01; 0.21]
Kim et al. (2022) [35] 0.212 300 0.21 [0.10; 0.32]
Kim & Kim (2022) [32] 0.210 1519 0.21 [0.16; 0.26]
Choi et al. (2024) [37] 0.270 47 0.27 [-0.02; 0.52]
Cho (2025) 0.108 138 0.11 [-0.06; 0.27]
Random effects model 0.18 [0.13; 0.22]
Heterogeneity: I^2 = 1.1%, τ^2 = 0.0007, p = 0.4089
h = Usual activities
Jo (2014) [14] 0.370 300 0.37 [0.27; 0.46]
Kim et al. (2021) [34] 0.249 300 0.25 [0.14; 0.35]
Kim et al. (2022) [35] 0.372 300 0.37 [0.27; 0.47]
Kim & Kim (2022) [32] 0.280 1519 0.28 [0.23; 0.33]
Choi et al. (2024) [37] 0.320 47 0.32 [0.04; 0.56]
Cho (2025) 0.170 138 0.17 [0.00; 0.33]
Random effects model 0.30 [0.24; 0.35]
Heterogeneity: I^2 = 36.5%, τ^2 = 0.0019, p = 0.1635
h = Pain/Discomfort
Jo (2014) [14] 0.424 300 0.42 [0.33; 0.51]
Kim et al. (2021) [34] 0.290 300 0.29 [0.18; 0.39]
Kim et al. (2022) [35] 0.344 300 0.34 [0.24; 0.44]
Kim & Kim (2022) [32] 0.240 1519 0.24 [0.19; 0.29]
Choi et al. (2024) [37] 0.538 47 0.54 [0.30; 0.71]
Cho (2025) 0.289 138 0.29 [0.13; 0.44]
Random effects model 0.33 [0.26; 0.40]
Heterogeneity: I^2 = 69.7%, τ^2 = 0.0060, p = 0.0055
h = Anxiety/Depression
Jo (2014) [14] 0.351 300 0.35 [0.25; 0.45]
Kim et al. (2021) [34] 0.331 300 0.33 [0.23; 0.43]
Kim et al. (2022) [35] 0.385 300 0.38 [0.28; 0.48]
Kim & Kim (2022) [32] 0.230 1519 0.23 [0.18; 0.28]
Choi et al. (2024) [37] 0.356 47 0.36 [0.08; 0.58]
Cho (2025) 0.226 138 0.23 [0.06; 0.38]
Random effects model 0.31 [0.24; 0.37]
Heterogeneity: I^2 = 58.4%, τ^2 = 0.0038, p = 0.0347
-0.4 -0.2 0 0.2 0.4 0.6 0.8 1
Correlation (r)

Working

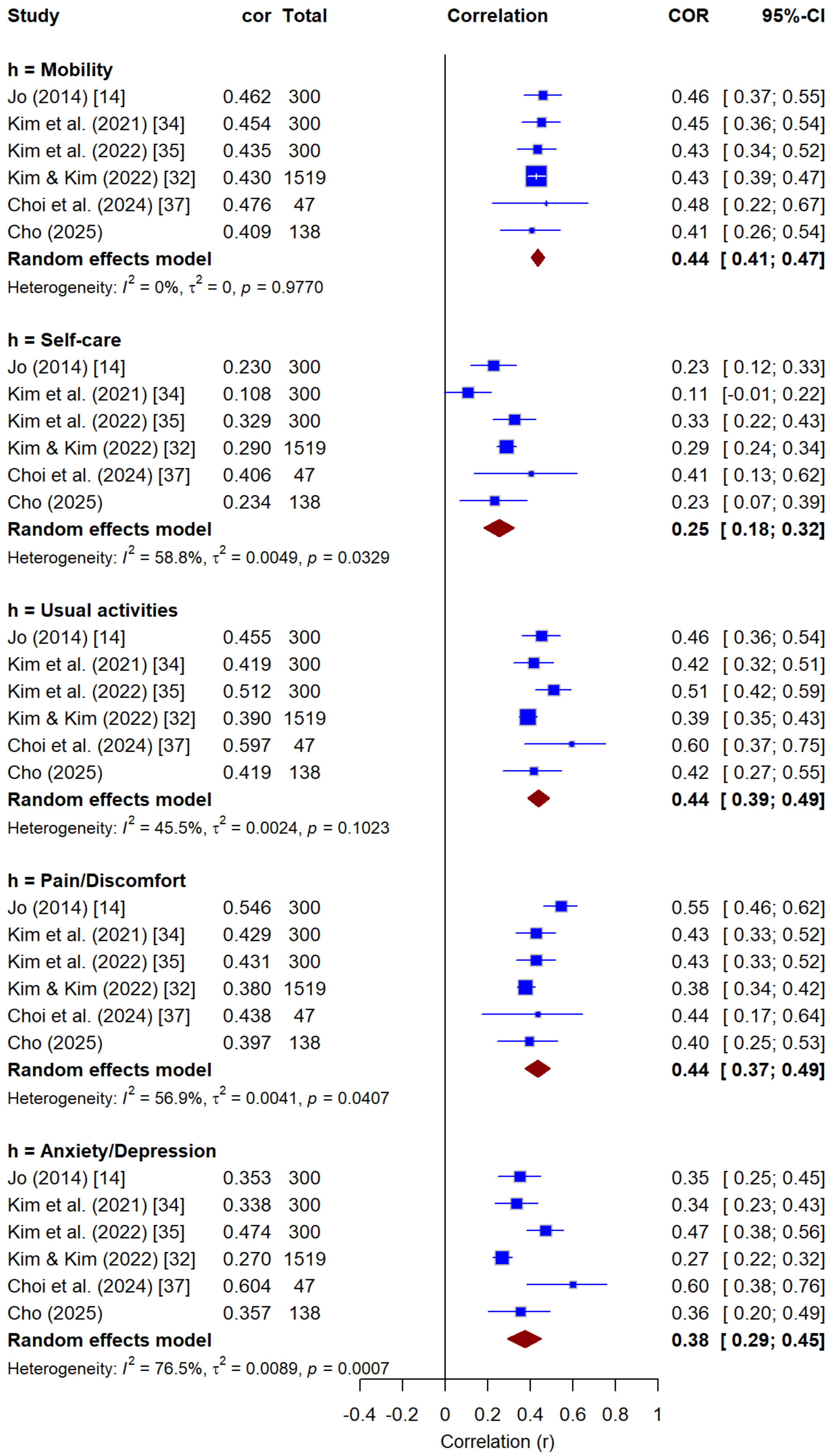
Study cor Total Correlation COR 95%-CI
h = Mobility
Jo (2014) [14] 0.462 300 0.46 [0.37; 0.55]
Kim et al. (2021) [34] 0.454 300 0.45 [0.36; 0.54]
Kim et al. (2022) [35] 0.435 300 0.43 [0.34; 0.52]
Kim & Kim (2022) [32] 0.430 1519 0.43 [0.39; 0.47]
Choi et al. (2024) [37] 0.476 47 0.48 [0.22; 0.67]
Cho (2025) 0.409 138 0.41 [0.26; 0.54]
Random effects model 0.44 [0.41; 0.47]
Heterogeneity: I^2 = 0%, τ^2 = 0, p = 0.9770
h = Self-care
Jo (2014) [14] 0.230 300 0.23 [0.12; 0.33]
Kim et al. (2021) [34] 0.108 300 0.11 [-0.01; 0.22]
Kim et al. (2022) [35] 0.329 300 0.33 [0.22; 0.43]
Kim & Kim (2022) [32] 0.290 1519 0.29 [0.24; 0.34]
Choi et al. (2024) [37] 0.406 47 0.41 [0.13; 0.62]
Cho (2025) 0.234 138 0.23 [0.07; 0.39]
Random effects model 0.25 [0.18; 0.32]
Heterogeneity: I^2 = 58.8%, τ^2 = 0.0049, p = 0.0329
h = Usual activities
Jo (2014) [14] 0.455 300 0.46 [0.36; 0.54]
Kim et al. (2021) [34] 0.419 300 0.42 [0.32; 0.51]
Kim et al. (2022) [35] 0.512 300 0.51 [0.42; 0.59]
Kim & Kim (2022) [32] 0.390 1519 0.39 [0.35; 0.43]
Choi et al. (2024) [37] 0.597 47 0.60 [0.37; 0.75]
Cho (2025) 0.419 138 0.42 [0.27; 0.55]
Random effects model 0.44 [0.39; 0.49]
Heterogeneity: I^2 = 45.5%, τ^2 = 0.0024, p = 0.1023
h = Pain/Discomfort
Jo (2014) [14] 0.546 300 0.55 [0.46; 0.62]
Kim et al. (2021) [34] 0.429 300 0.43 [0.33; 0.52]
Kim et al. (2022) [35] 0.431 300 0.43 [0.33; 0.52]
Kim & Kim (2022) [32] 0.380 1519 0.38 [0.34; 0.42]
Choi et al. (2024) [37] 0.438 47 0.44 [0.17; 0.64]
Cho (2025) 0.397 138 0.40 [0.25; 0.53]
Random effects model 0.44 [0.37; 0.49]
Heterogeneity: I^2 = 56.9%, τ^2 = 0.0041, p = 0.0407
h = Anxiety/Depression
Jo (2014) [14] 0.353 300 0.35 [0.25; 0.45]
Kim et al. (2021) [34] 0.338 300 0.34 [0.23; 0.43]
Kim et al. (2022) [35] 0.474 300 0.47 [0.38; 0.56]
Kim & Kim (2022) [32] 0.270 1519 0.27 [0.22; 0.32]
Choi et al. (2024) [37] 0.604 47 0.60 [0.38; 0.76]
Cho (2025) 0.357 138 0.36 [0.20; 0.49]
Random effects model 0.38 [0.29; 0.45]
Heterogeneity: I^2 = 76.5%, τ^2 = 0.0089, p = 0.0007
-0.4 -0.2 0 0.2 0.4 0.6 0.8 1
Correlation (r)

Depression

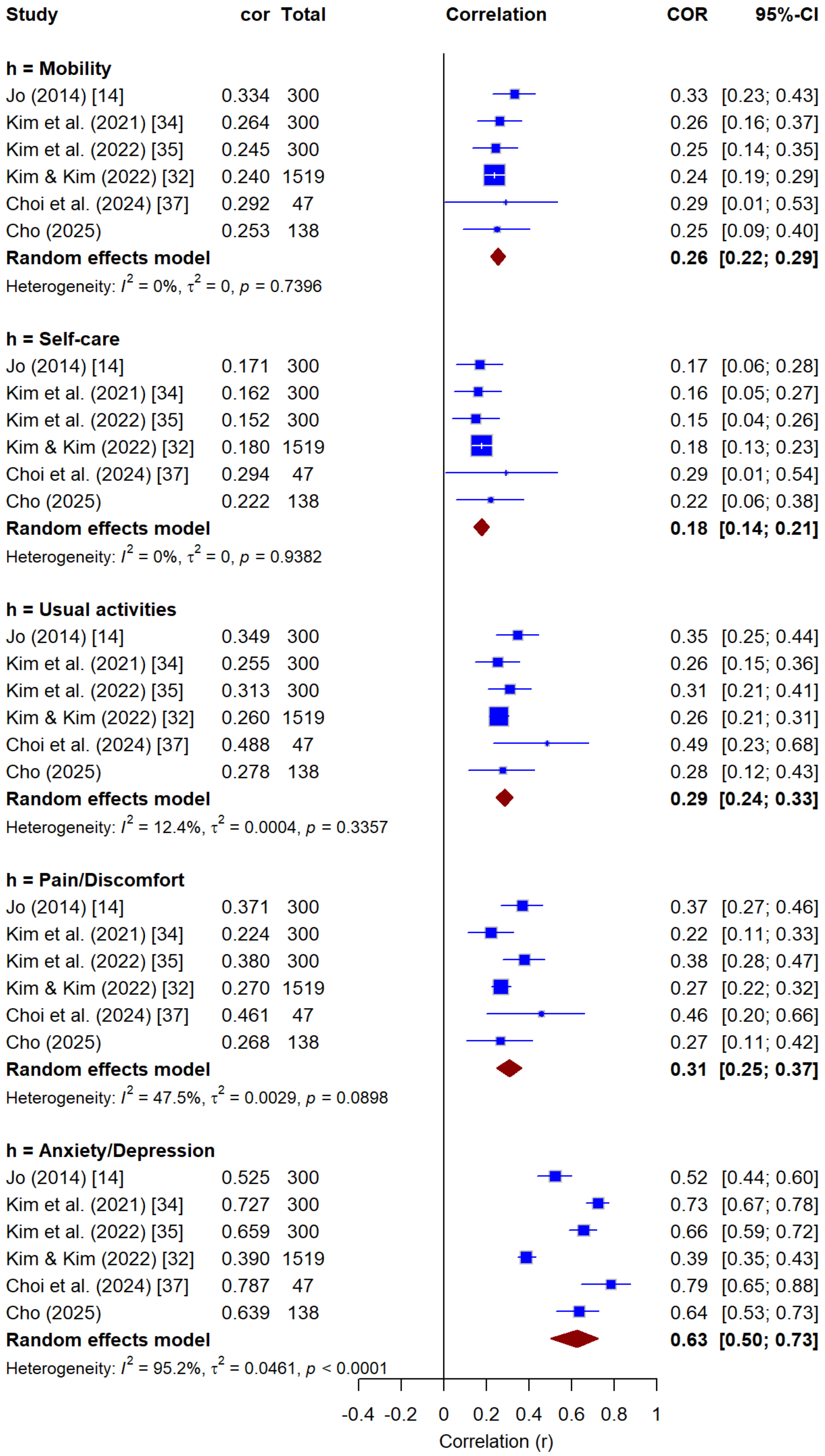
Study cor Total Correlation COR 95%-CI
h = Mobility
Jo (2014) [14] 0.334 300 0.33 [0.23; 0.43]
Kim et al. (2021) [34] 0.264 300 0.26 [0.16; 0.37]
Kim et al. (2022) [35] 0.245 300 0.25 [0.14; 0.35]
Kim & Kim (2022) [32] 0.240 1519 0.24 [0.19; 0.29]
Choi et al. (2024) [37] 0.292 47 0.29 [0.01; 0.53]
Cho (2025) 0.253 138 0.25 [0.09; 0.40]
Random effects model 0.26 [0.22; 0.29]
Heterogeneity: $I^2 = 0\%$, $\tau^2 = 0$, $p = 0.7396$
h = Self-care
Jo (2014) [14] 0.171 300 0.17 [0.06; 0.28]
Kim et al. (2021) [34] 0.162 300 0.16 [0.05; 0.27]
Kim et al. (2022) [35] 0.152 300 0.15 [0.04; 0.26]
Kim & Kim (2022) [32] 0.180 1519 0.18 [0.13; 0.23]
Choi et al. (2024) [37] 0.294 47 0.29 [0.01; 0.54]
Cho (2025) 0.222 138 0.22 [0.06; 0.38]
Random effects model 0.18 [0.14; 0.21]
Heterogeneity: $I^2 = 0\%$, $\tau^2 = 0$, $p = 0.9382$
h = Usual activities
Jo (2014) [14] 0.349 300 0.35 [0.25; 0.44]
Kim et al. (2021) [34] 0.255 300 0.26 [0.15; 0.36]
Kim et al. (2022) [35] 0.313 300 0.31 [0.21; 0.41]
Kim & Kim (2022) [32] 0.260 1519 0.26 [0.21; 0.31]
Choi et al. (2024) [37] 0.488 47 0.49 [0.23; 0.68]
Cho (2025) 0.278 138 0.28 [0.12; 0.43]
Random effects model 0.29 [0.24; 0.33]
Heterogeneity: $I^2 = 12.4\%$, $\tau^2 = 0.0004$, $p = 0.3357$
h = Pain/Discomfort
Jo (2014) [14] 0.371 300 0.37 [0.27; 0.46]
Kim et al. (2021) [34] 0.224 300 0.22 [0.11; 0.33]
Kim et al. (2022) [35] 0.380 300 0.38 [0.28; 0.47]
Kim & Kim (2022) [32] 0.270 1519 0.27 [0.22; 0.32]
Choi et al. (2024) [37] 0.461 47 0.46 [0.20; 0.66]
Cho (2025) 0.268 138 0.27 [0.11; 0.42]
Random effects model 0.31 [0.25; 0.37]
Heterogeneity: $I^2 = 47.5\%$, $\tau^2 = 0.0029$, $p = 0.0898$
h = Anxiety/Depression
Jo (2014) [14] 0.525 300 0.52 [0.44; 0.60]
Kim et al. (2021) [34] 0.727 300 0.73 [0.67; 0.78]
Kim et al. (2022) [35] 0.659 300 0.66 [0.59; 0.72]
Kim & Kim (2022) [32] 0.390 1519 0.39 [0.35; 0.43]
Choi et al. (2024) [37] 0.787 47 0.79 [0.65; 0.88]
Cho (2025) 0.639 138 0.64 [0.53; 0.73]
Random effects model 0.63 [0.50; 0.73]
Heterogeneity: $I^2 = 95.2\%$, $\tau^2 = 0.0461$, $p < 0.0001$
-0.4 -0.2 0 0.2 0.4 0.6 0.8 1
Correlation (r)

Memory

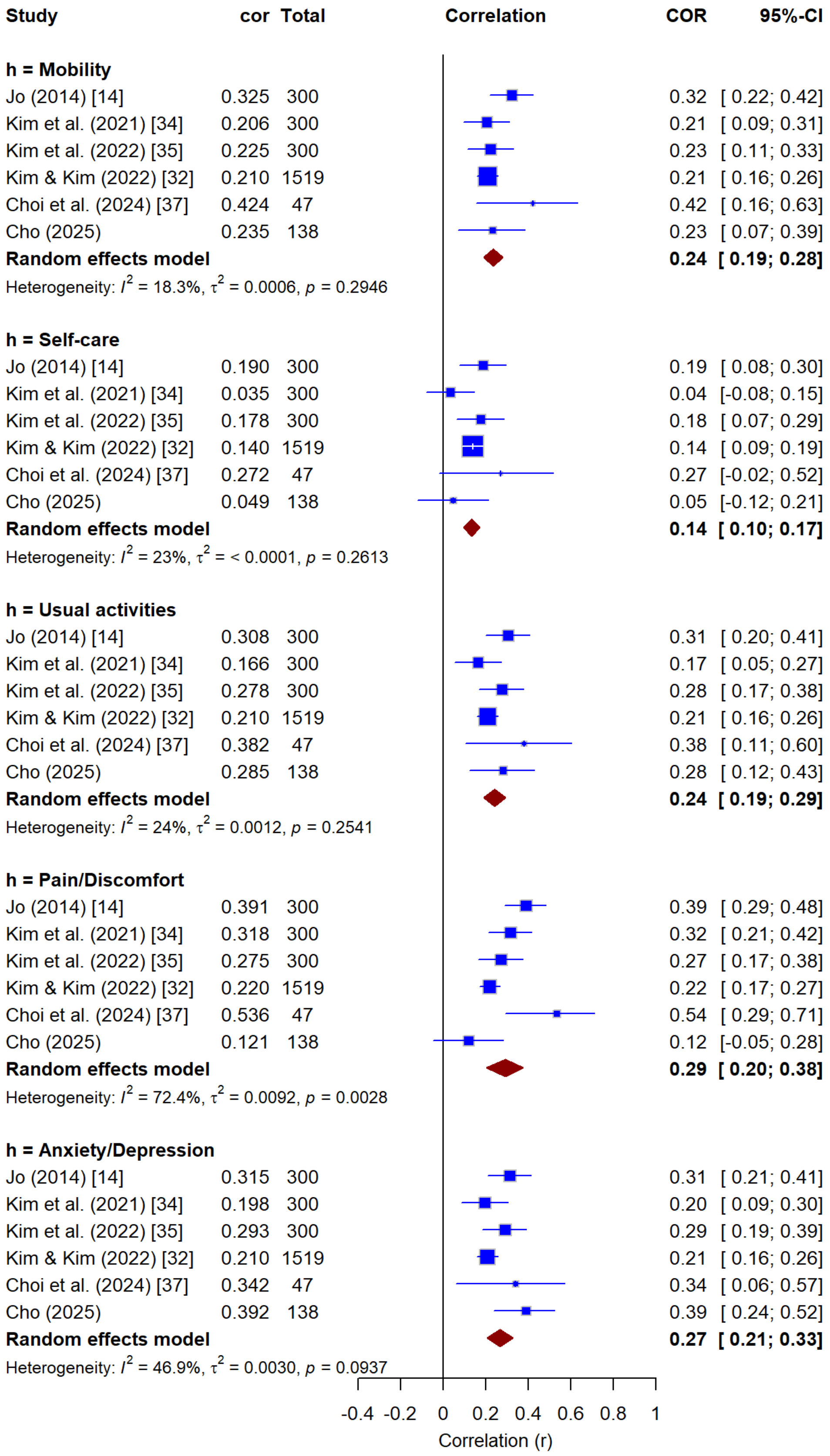

Study cor Total Correlation COR 95%-CI
h = Mobility
Jo (2014) [14] 0.325 300 0.32 [0.22; 0.42]
Kim et al. (2021) [34] 0.206 300 0.21 [0.09; 0.31]
Kim et al. (2022) [35] 0.225 300 0.23 [0.11; 0.33]
Kim & Kim (2022) [32] 0.210 1519 0.21 [0.16; 0.26]
Choi et al. (2024) [37] 0.424 47 0.42 [0.16; 0.63]
Cho (2025) 0.235 138 0.23 [0.07; 0.39]
Random effects model 0.24 [0.19; 0.28]
Heterogeneity: I^2 = 18.3%, τ^2 = 0.0006, p = 0.2946
h = Self-care
Jo (2014) [14] 0.190 300 0.19 [0.08; 0.30]
Kim et al. (2021) [34] 0.035 300 0.04 [-0.08; 0.15]
Kim et al. (2022) [35] 0.178 300 0.18 [0.07; 0.29]
Kim & Kim (2022) [32] 0.140 1519 0.14 [0.09; 0.19]
Choi et al. (2024) [37] 0.272 47 0.27 [-0.02; 0.52]
Cho (2025) 0.049 138 0.05 [-0.12; 0.21]
Random effects model 0.14 [0.10; 0.17]
Heterogeneity: I^2 = 23%, τ^2 = < 0.0001, p = 0.2613
h = Usual activities
Jo (2014) [14] 0.308 300 0.31 [0.20; 0.41]
Kim et al. (2021) [34] 0.166 300 0.17 [0.05; 0.27]
Kim et al. (2022) [35] 0.278 300 0.28 [0.17; 0.38]
Kim & Kim (2022) [32] 0.210 1519 0.21 [0.16; 0.26]
Choi et al. (2024) [37] 0.382 47 0.38 [0.11; 0.60]
Cho (2025) 0.285 138 0.28 [0.12; 0.43]
Random effects model 0.24 [0.19; 0.29]
Heterogeneity: I^2 = 24%, τ^2 = 0.0012, p = 0.2541
h = Pain/Discomfort
Jo (2014) [14] 0.391 300 0.39 [0.29; 0.48]
Kim et al. (2021) [34] 0.318 300 0.32 [0.21; 0.42]
Kim et al. (2022) [35] 0.275 300 0.27 [0.17; 0.38]
Kim & Kim (2022) [32] 0.220 1519 0.22 [0.17; 0.27]
Choi et al. (2024) [37] 0.536 47 0.54 [0.29; 0.71]
Cho (2025) 0.121 138 0.12 [-0.05; 0.28]
Random effects model 0.29 [0.20; 0.38]
Heterogeneity: I^2 = 72.4%, τ^2 = 0.0092, p = 0.0028
h = Anxiety/Depression
Jo (2014) [14] 0.315 300 0.31 [0.21; 0.41]
Kim et al. (2021) [34] 0.198 300 0.20 [0.09; 0.30]
Kim et al. (2022) [35] 0.293 300 0.29 [0.19; 0.39]
Kim & Kim (2022) [32] 0.210 1519 0.21 [0.16; 0.26]
Choi et al. (2024) [37] 0.342 47 0.34 [0.06; 0.57]
Cho (2025) 0.392 138 0.39 [0.24; 0.52]
Random effects model 0.27 [0.21; 0.33]
Heterogeneity: I^2 = 46.9%, τ^2 = 0.0030, p = 0.0937
-0.4 -0.2 0 0.2 0.4 0.6 0.8 1
Correlation (r)

Sleep

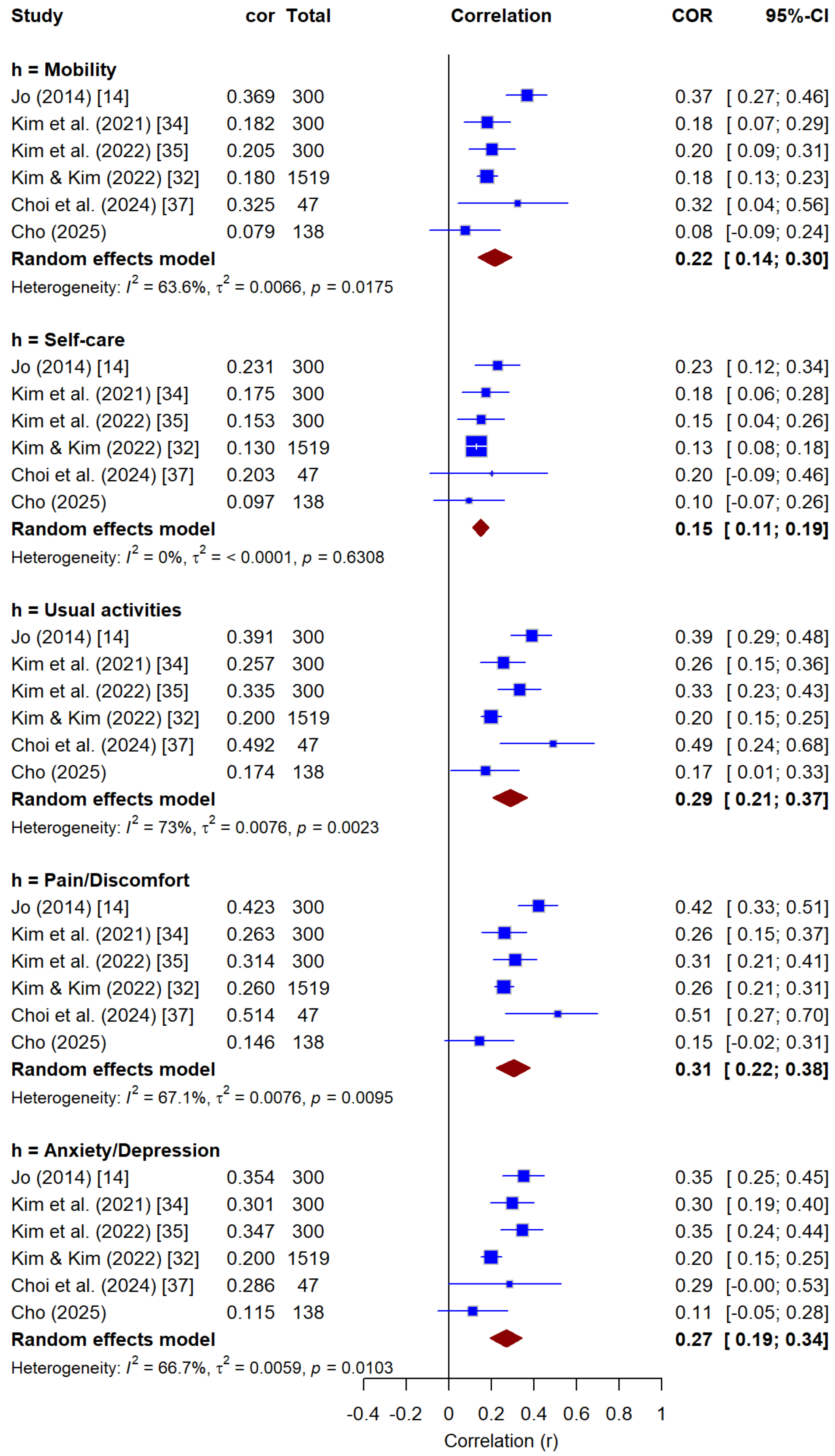

Study cor Total Correlation COR 95%-CI
h = Mobility
Jo (2014) [14] 0.369 300 0.37 [0.27; 0.46]
Kim et al. (2021) [34] 0.182 300 0.18 [0.07; 0.29]
Kim et al. (2022) [35] 0.205 300 0.20 [0.09; 0.31]
Kim & Kim (2022) [32] 0.180 1519 0.18 [0.13; 0.23]
Choi et al. (2024) [37] 0.325 47 0.32 [0.04; 0.56]
Cho (2025) 0.079 138 0.08 [-0.09; 0.24]
Random effects model 0.22 [0.14; 0.30]
Heterogeneity: I^2 = 63.6%, τ^2 = 0.0066, p = 0.0175
h = Self-care
Jo (2014) [14] 0.231 300 0.23 [0.12; 0.34]
Kim et al. (2021) [34] 0.175 300 0.18 [0.06; 0.28]
Kim et al. (2022) [35] 0.153 300 0.15 [0.04; 0.26]
Kim & Kim (2022) [32] 0.130 1519 0.13 [0.08; 0.18]
Choi et al. (2024) [37] 0.203 47 0.20 [-0.09; 0.46]
Cho (2025) 0.097 138 0.10 [-0.07; 0.26]
Random effects model 0.15 [0.11; 0.19]
Heterogeneity: I^2 = 0%, τ^2 = < 0.0001, p = 0.6308
h = Usual activities
Jo (2014) [14] 0.391 300 0.39 [0.29; 0.48]
Kim et al. (2021) [34] 0.257 300 0.26 [0.15; 0.36]
Kim et al. (2022) [35] 0.335 300 0.33 [0.23; 0.43]
Kim & Kim (2022) [32] 0.200 1519 0.20 [0.15; 0.25]
Choi et al. (2024) [37] 0.492 47 0.49 [0.24; 0.68]
Cho (2025) 0.174 138 0.17 [0.01; 0.33]
Random effects model 0.29 [0.21; 0.37]
Heterogeneity: I^2 = 73%, τ^2 = 0.0076, p = 0.0023
h = Pain/Discomfort
Jo (2014) [14] 0.423 300 0.42 [0.33; 0.51]
Kim et al. (2021) [34] 0.263 300 0.26 [0.15; 0.37]
Kim et al. (2022) [35] 0.314 300 0.31 [0.21; 0.41]
Kim & Kim (2022) [32] 0.260 1519 0.26 [0.21; 0.31]
Choi et al. (2024) [37] 0.514 47 0.51 [0.27; 0.70]
Cho (2025) 0.146 138 0.15 [-0.02; 0.31]
Random effects model 0.31 [0.22; 0.38]
Heterogeneity: I^2 = 67.1%, τ^2 = 0.0076, p = 0.0095
h = Anxiety/Depression
Jo (2014) [14] 0.354 300 0.35 [0.25; 0.45]
Kim et al. (2021) [34] 0.301 300 0.30 [0.19; 0.40]
Kim et al. (2022) [35] 0.347 300 0.35 [0.24; 0.44]
Kim & Kim (2022) [32] 0.200 1519 0.20 [0.15; 0.25]
Choi et al. (2024) [37] 0.286 47 0.29 [-0.00; 0.53]
Cho (2025) 0.115 138 0.11 [-0.05; 0.28]
Random effects model 0.27 [0.19; 0.34]
Heterogeneity: I^2 = 66.7%, τ^2 = 0.0059, p = 0.0103
-0.4 -0.2 0 0.2 0.4 0.6 0.8 1
Correlation (r)

Happiness

Study	cor	Total	COR	95%-CI
**h = Mobility**				
Jo (2014) [14]	0.256	300	0.26	[0.15; 0.36]
Kim et al. (2021) [34]	0.246	300	0.25	[0.14; 0.35]
Kim et al. (2022) [35]	0.218	300	0.22	[0.11; 0.32]
Kim & Kim (2022) [32]	0.250	1519	0.25	[0.20; 0.30]
Choi et al. (2024) [37]	0.111	47	0.11	[-0.18; 0.39]
Cho (2025)	0.153	138	0.15	[-0.01; 0.31]
**Random effects model**			**0.24**	**[0.20; 0.28]**
Heterogeneity: $I^2 = 0\%$, $\tau^2 = 0$, $p = 0.8045$				
**h = Self-care**				
Jo (2014) [14]	0.035	300	0.04	[-0.08; 0.15]
Kim et al. (2021) [34]	0.071	300	0.07	[-0.04; 0.18]
Kim et al. (2022) [35]	0.244	300	0.24	[0.13; 0.35]
Kim & Kim (2022) [32]	0.200	1519	0.20	[0.15; 0.25]
Choi et al. (2024) [37]	-0.133	47	-0.13	[-0.40; 0.16]
Cho (2025)	0.168	138	0.17	[0.00; 0.33]
**Random effects model**			**0.13**	**[0.04; 0.21]**
Heterogeneity: $I^2 = 68.4\%$, $\tau^2 = 0.0070$, $p = 0.0074$				
**h = Usual activities**				
Jo (2014) [14]	0.245	300	0.25	[0.14; 0.35]
Kim et al. (2021) [34]	0.229	300	0.23	[0.12; 0.33]
Kim et al. (2022) [35]	0.351	300	0.35	[0.25; 0.45]
Kim & Kim (2022) [32]	0.270	1519	0.27	[0.22; 0.32]
Choi et al. (2024) [37]	0.363	47	0.36	[0.08; 0.59]
Cho (2025)	0.219	138	0.22	[0.05; 0.37]
**Random effects model**			**0.27**	**[0.23; 0.31]**
Heterogeneity: $I^2 = 0\%$, $\tau^2 = 0$, $p = 0.5440$				
**h = Pain/Discomfort**				
Jo (2014) [14]	0.320	300	0.32	[0.21; 0.42]
Kim et al. (2021) [34]	0.243	300	0.24	[0.13; 0.35]
Kim et al. (2022) [35]	0.392	300	0.39	[0.29; 0.48]
Kim & Kim (2022) [32]	0.230	1519	0.23	[0.18; 0.28]
Choi et al. (2024) [37]	0.043	47	0.04	[-0.25; 0.33]
Cho (2025)	0.258	138	0.26	[0.09; 0.41]
**Random effects model**			**0.27**	**[0.21; 0.34]**
Heterogeneity: $I^2 = 57\%$, $\tau^2 = 0.0037$, $p = 0.0404$				
**h = Anxiety/Depression**				
Jo (2014) [14]	0.313	300	0.31	[0.21; 0.41]
Kim et al. (2021) [34]	0.511	300	0.51	[0.42; 0.59]
Kim et al. (2022) [35]	0.563	300	0.56	[0.48; 0.64]
Kim & Kim (2022) [32]	0.310	1519	0.31	[0.26; 0.35]
Choi et al. (2024) [37]	0.306	47	0.31	[0.02; 0.55]
Cho (2025)	0.483	138	0.48	[0.34; 0.60]
**Random effects model**			**0.43**	**[0.32; 0.52]**
Heterogeneity: $I^2 = 87\%$, $\tau^2 = 0.0175$, $p < 0.0001$				

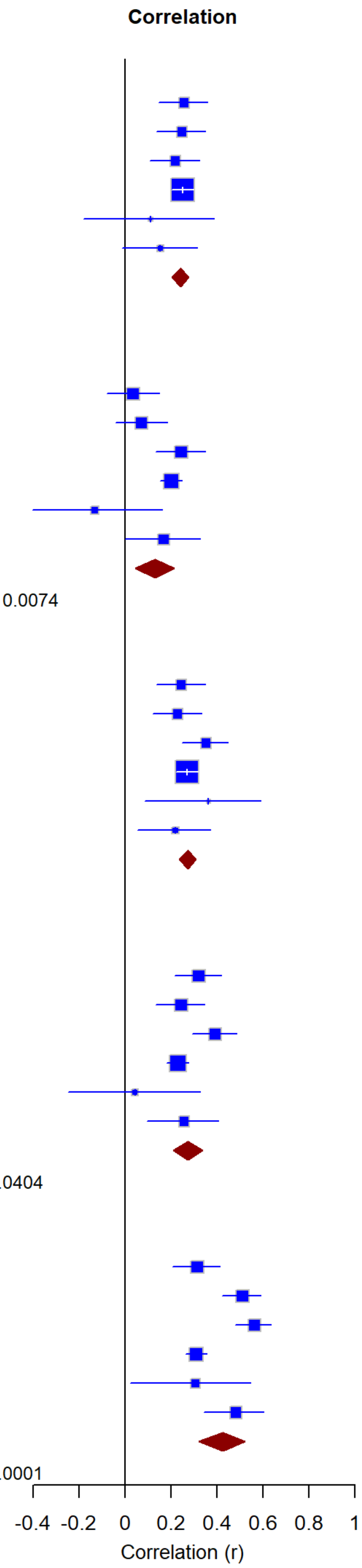

HINT-8 index

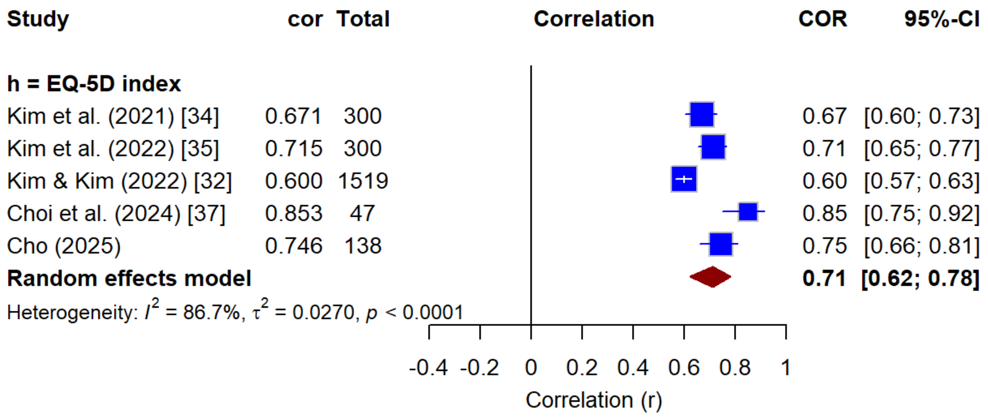

Note. Cho (2025) is additional unpublished data from our research group.
