# Supplementary material for: Psychometric properties of the health-related quality of life instrument with 8 items: a systematic review and meta-analysis
Source: Health Qual Life Outcomes. 2026 Mar 4;24:47. doi: 10.1186/s12955-026-02494-z (PMC13067613; doi:10.1186/s12955-026-02494-z)
Supplement: Supplementary file 9 — Supplementary Material 9 [file 12955_2026_2494_MOESM9_ESM.pdf]

Supplementary Material 9. Pooled HINT-8 index for specific diseases and health status compared with the EQ-5D-3L index from 12 studies

Type of disease	Pooled HINT-8 index	EQ-5D-3L mean index	Studies reporting EQ-5D scores
Generalized anxiety disorder	0.562	-	-
Restriction of activity	0.645	-	-
Dizziness	0.660	-	-
Depression	0.672	0.861 and 0.863 (by gender)	Cho et al. (2019) [43]
Poor subjective health status	0.700	-	-
Arthritis	0.701	0.84 (osteoarthritis with activity limitation)	Lee & Kim (2020) [44]
		0.849	Chae et al. (2023) [45]
		0.73 (osteoarthritis)	Lim et al. (2010) [46]
High-stress	0.704	-	-
Dyslipidemia	0.745	0.910	Chae et al. (2023) [45]
Type 2 diabetes	0.746	0.898	
Hypertension	0.747	0.900	
Cancer	0.750	0.70-0.92 (30 different cancer types)	Kim et al. (2012) [49]
Gastroesophageal reflux disease	0.759	[a]0.72	Park et al. (2019) [41]
		0.88 (with laryngopharyngeal reflux)	Gong et al. (2017) [50]
Chronic cardiovascular disease	0.763	[b]0.871	Chung et al. (2024) [38]
		0.85	Kim & Kim (2022) [32]
		0.831 (MI, angina pectoris)	Lee et al. (2015) [47]
Chronic respiratory disease	0.790	[b]0.794	Chung et al. (2024) [38]
		0.873 (asthma)	Chae et al. (2023) [45]
		0.933 (COPD)	Kim & Kim (2022) [33]
		0.87 (bronchiectasis)<br>0.89 (COPD)	Yang et al. (2019) [48]

Notes: [a]EQ-5D version not specified; [b]EQ-5D-5L, mean scores were calculated from reported median scores. COPD=chronic obstructive pulmonary disease; MI=myocardial infarction
